# Breast and Bottle Feeding as Risk Factors for Dental Caries: A Systematic Review and Meta-Analysis

**DOI:** 10.1371/journal.pone.0142922

**Published:** 2015-11-18

**Authors:** Walesca M. Avila, Isabela A. Pordeus, Saul M. Paiva, Carolina C. Martins

**Affiliations:** Department of Pediatric Dentistry and Orthodontics, Faculty of Dentistry, Universidade Federal de Minas Gerais, Belo Horizonte, Minas Gerais, Brazil; Canadian Agency for Drugs and Technologies in Health, CANADA

## Abstract

Understanding the role that breastfeeding and bottle feeding play in the development of dental caries during childhood is essential in helping dentists and parents and care providers prevent the disease, and also for the development of effective public health policies. However, the issue is not yet fully understood. The aim of this systematic review and meta-analysis was to search for scientific evidence in response to the question: Do bottle fed children have more dental caries in primary dentition than breastfed children? Seven electronic databases and grey literature were used in the search. The protocol number of the study is PROSPERO CRD 42014006534. Two independent reviewers selected the studies, extracted data and evaluated risk of bias by quality assessment. A random effect model was used for meta-analysis, and the summary effect measure were calculated by odds ratio (OR) and 95% CI. Seven studies were included: five cross-sectional, one case-control and one cohort study. A meta-analysis of cross-sectional studies showed that breastfed children were less affected by dental caries than bottle fed children (OR: 0.43; 95%CI: 0.23–0.80). Four studies showed that bottle fed children had more dental caries (p<0.05), while three studies found no such association (p>0.05). The scientific evidence therefore indicated that breastfeeding can protect against dental caries in early childhood. The benefits of breastfeeding until age two is recommended by WHO/UNICEF guidelines. Further prospective observational cohort studies are needed to strengthen the evidence.

## Introduction

Early childhood caries (ECC) are defined as the presence of one or more decayed, missing or filled tooth surface in any primary tooth of children aged under 71 months [1].

One of the first published reports into dental caries in babies was performed in 1927[2], when doctors noticed that a large number of babies had extensive caries in tooth surfaces. Although no research into the role of breastfeeding and bottle feeding in the etiology of ECC existed at this time, many studies since then have revealed ambiguous results with respect to feeding habits and dental caries [3, 4].

The benefits of breastfeeding for systemic health, such as the reduction of morbidity, infectious disease and low weight in newborns [5], are well known. The PROBIT trial emphasized the importance of breastfeeding, as it decreased the risk of gastrointestinal infections and inflammatory skin conditions [6]. Although it seems the practice does not benefit the development of normal occlusion [7]. Exclusive breastfeeding is recommended by the World Health Organization (WHO) until the age of six months, and breastfeeding complemented with food intake is suggested until two years old [8]. However, cultural and social factors directly affect knowledge of how long a child should be breastfed for [9].

The issue of whether bottle feeding is more cariogenic than breastfeeding remains unresolved even today. Some authors have not found an association between breastfeeding and dental caries [10–12], while other study have reported the existence of such an association[13]. Some authors have stated that bottle feeding is a risk factor for dental caries [14–16], while another author did not find such an association [17]. Due to the disagreement between these findings, further studies are needed to clarify the existence of this association [18].

A systematic review of studies investigating the relationship between breastfeeding and dental caries was published in 2000 and included twenty four case-control studies, three case series and one cohort. The systematic review could not confirm that breastfeeding was a risk factor of dental caries. However, it did not report comparisons between breastfeeding and bottle feeding [19]. Another review [20] identified three factors related to breastfeeding and/or bottle feeding as risk factors for dental caries: duration of breastfeeding greater than 18 months, used to feed or stop crying during the night, and to put the child to sleep. However, none of these reviews compared bottle feeding vs. breastfeeding in relation to dental caries, and as such it has not been confirmed whether bottle feeding is more associated with dental caries in primary dentition than is breast feeding. Fifteen years later, the issue of whether bottle feeding can contribute to an increased risk of dental caries compared to breastfeeding remains unclear, as none of the reviews aimed to answer this clinical question. Therefore, this systematic review is the first to compare the rate of caries in different type of feeding practices: breastfeeding and bottle feeding.

Greater understanding of the subject is important, however, as improved knowledge can help dentists provide more appropriate instructions and lead to healthier children. The presence of dental caries in childhood is an important theme, which should be exhaustively discussed and treated as it affects well-being, growth [21] and quality of life [22]. Despite a decrease in the prevalence of dental caries in both developed [23] and developing countries [24], worldwide prevalence in five-year-old children remains high, with a level of 27.9% in England [25]; 46.6% in Brazil [26], between 11.0 and 53.0% in the USA [27] and 23% in American children aged 2–3 years old [28].

The aim of this study was to systematically review the scientific evidence relating to the association between feeding practice (breastfeeding vs. bottle feeding) and dental caries in childhood. The clinical question is (PICO): Patients: children with exclusively primary dentition; Intervention / Exposure to risk factor: bottle feeding; Comparison: breast feeding; Outcome: dental caries.

## Material and Methods

The present systematic review was undertaken in accordance with the guidelines of the Preferred Reporting Items for Systematic Reviews and Meta-Analyses (PRISMA) [29] (protocol number: PROSPERO CRD 42014006534).

This systematic review included observational cross-sectional, case-control, and cohort studies, together with clinical trials of children with exclusively primary dentition (age ≤ 71 months), which compared breastfeeding and bottle feeding in association with dental caries, and included statistical data comparing bottle to breast feeding. Statistical data could be: *odds ratio* (OR), *relative risk* (RR), *prevalence ratio* (PR), confidence intervals (95%CI), p-values, or studies that reported frequency or an absolute number of events/total number of individuals per group.

Seven electronic databases were searched in March 2014: Pubmed (www.pubmed.gov); Cochrane Library (http://www.cochrane.org/index.html); Web of Science (http://www.isiknowledge.com); Controlled-trials Database of Clinical Trials (http://www.controlled-trials.com); Clinical Trials–US National Institute of Health (http://www.clinicaltrials.gov); National Institute for Health and Clinical Excellence (http://www.nice.org.uk); Lilacs (www.bireme.br) without restriction of date of publication. The search was updated in March 2015.

The following search strategy was used for the Pubmed, Cochrane Library and Web of Science databases: ((caries OR dental caries OR dental decay OR decay OR DMF index OR DMF Indices OR decayed teeth OR tooth decay) AND (bottle feeding OR bottlefeedings OR bottlefeed* OR breastfed* OR breast fed OR breastfeeding)).

The controlled-trials Database of Clinical Trials, Clinical Trials, National Institute for Health and Clinical Excellence, Lilacs were searched using the following combined keywords: dental caries AND breast feeding AND bottle feeding. A manual search was conducted in the reference lists of the included studies.

**Fig 1:**
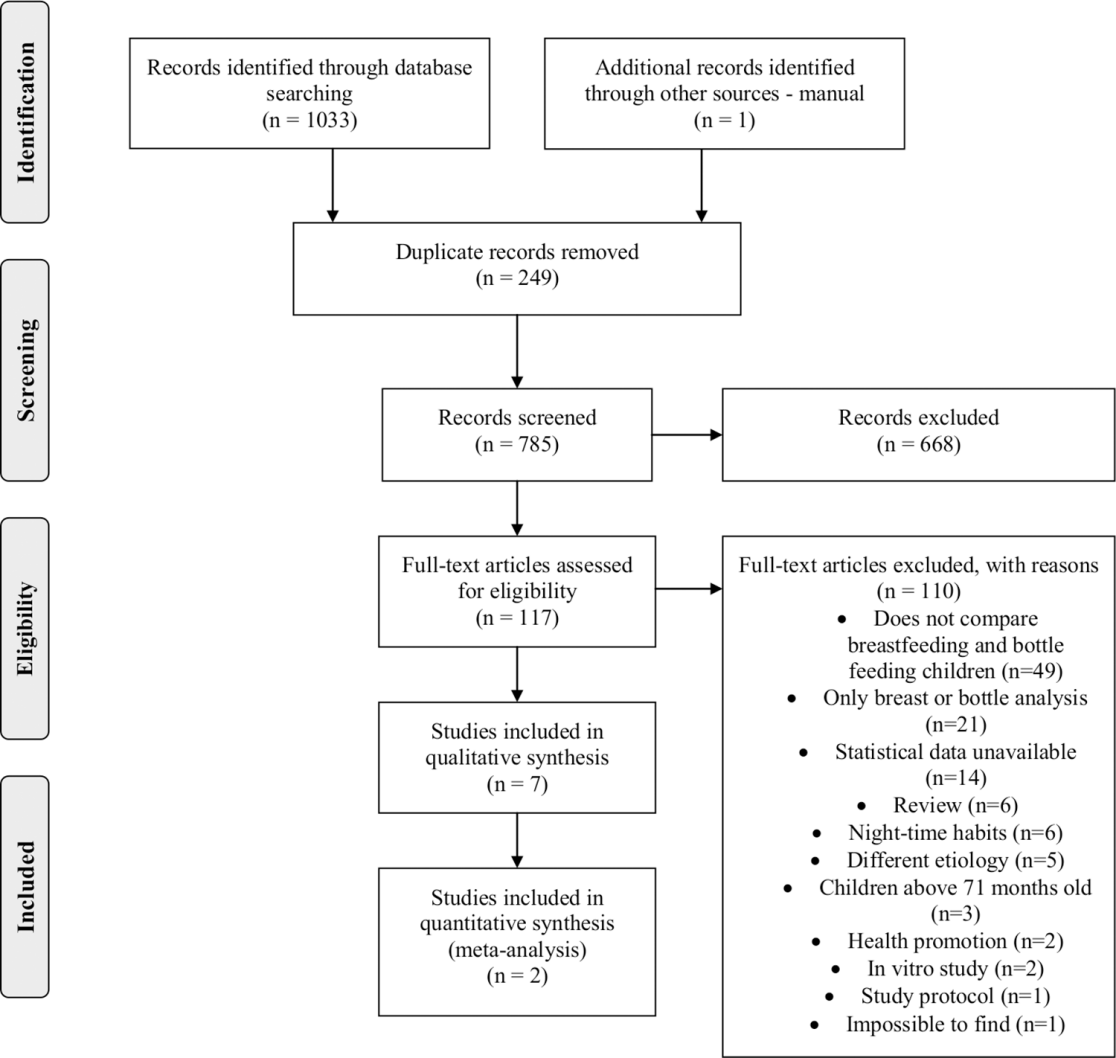
Screening of articles. Four-phase PRISMA flow-diagram for study collection, showing number of studies identified, screened, eligible, included in review and meta-analysis [26].

The online search identified a total of 1033 papers (Fig 1). After duplicate references were removed, a total of 784 studies were entered in the Reference Manager^®^ program (Reference Manager, Thomson Reuters, version 12.0.3). The list provided by the reference manager was analyzed, and articles were selected based on abstracts and/or title by two independent reviewers (WMA and an undergraduate student). The independent reviewers were calibrated in accordance with inclusion/exclusion criteria using a sample of 20% of the retrieved studies, and agreement between reviewers was found to be good (K = 0.79). The inclusion and exclusion criteria were applied independently to the remainder of the studies and any disagreement was resolved by consensus with a third reviewer (CCM).

The exclusion criteria were: literature review, letters to the editor, editorials, patient handout, case report or case series, in vitro studies, etiology other than breast or bottle feeding, treatment of dental caries, health promotion, outcome other than dental caries (eg. malocclusion, dental hypoplasia, and others), other feeding habits, study protocol, studies reporting only bottle or breastfeeding, animal studies, studies of quality of life, language other than English.

A total of 667 studies were excluded after title/abstract analysis and 117 were selected for full text analysis. Where the studies could not be found, authors were personally contacted by e-mail (for a list of excluded abstracts and/or title, see S1 Appendix). After full text analysis, 109 studies were excluded (for a list of excluded studies, see S2 Appendix). These studies were excluded for several reasons, such as: investigation of only one type of feeding practice (only breastfeeding or only bottle feeding), absence of comparison of breastfeeding and bottle feeding, investigation of other issues such as night-time feeding or weaning time, absence of statistical data, other etiology, in vitro study, case report, children above 71 months old. Grey literature was searched using abstracts presented in meetings, and a manual search was conducted from a reference list of included studies.

### Data extraction

Descriptive data of clinical and methodological factors such as country, local setting, initial and final sample, dental examination, feeding habit evaluation, statistics, outcome and study design were extracted. In case of missing or misunderstood data, the authors were personally contacted by e-mail.

### Methodological quality assessment

**Fig 2:**
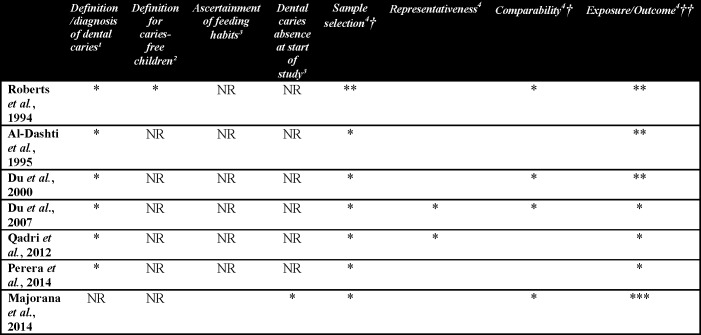
Newcastle-Ottawa quality assessment summary. ^1^For cross-sectional and case-control studies. ^2^For case-control study only. ^3^For cohort study only. ^4^For all study designs. †This item was allocated a maximum of 2 points. †† This item was allocated a maximum of 2 point for cross-sectional and 3 points for cohort and case-control studies. NR = not rated.

Quality assessment was performed by using the Newcastle-Ottawa Scale [30], which measures the methodological quality of a study by the number of points the study received. For case-control and cohort studies, the original scale was used. For cross-sectional studies, a modified version of the case-control study scale was used (Fig 2). Risk of bias was evaluated for each question. For each question-based entry the judgment was: “Yes, for low risk of bias” and a point was allocated (*), and “No, for high risk of bias” and a point was not allocated [31]. The questions evaluated in each study were based on the following criteria from the Newcastle-Ottawa scale: exposition/non-exposition and case/control definition; representativeness of the sample (evaluated by the methods of generation of samples, allocation concealment and sample calculation); sample selection (e.g., community, hospital, etc.), adjustment for confounders, blindness, acquisition of data on the dependent variable, description of bias, non-response rate (Fig 2).

The representativeness criteria was evaluated through the sampling methods. The presence of a random component in the sequence generation was judged as low risk of bias. Allocation concealment was also used as a criteria for assessing representativeness. Thus, any method that precluded participants and researchers from foreseeing assignment was judged as low risk of bias.

### Data synthesis

The Comprehensive Meta-Analysis software program (version 2) was used for meta-analysis [32]. Only studies with similar designs were included in the forest plot, as meta-analysis can provide misleading results if different study designs and studies variations across studies are grouped together [33]. For this reason, in order to avoid methodological heterogeneity in meta-analysis, only cross-sectional studies were grouped. Heterogeneity among the studies was evaluated using I^2^ statistics and a sensitivity test was used to test consistency of data by removing outliers from time series [33]. Two outliers caused statistical heterogeneity and had to be removed from the forest plot [11, 12]. Fixed effect model was used for low heterogeneity and random effect model for high heterogeneity. As values exceeding 50% can be considered to be of notable heterogeneity [34], the random effect model was used for these cases. [35]. For categorical data, risk measures, odds ratio (OR), 95% confidence intervals (CI) and p-values were calculated in a forest plot.

The studies featured different weaning ages or breastfeeding duration, different study designs and differences in statistical tests. Meta-analysis was conducted only for those studies featuring variables that could be grouped [11, 12, 36, 37]. It was not possible to extract data for meta-analysis for one cross-sectional study [16]. Data was extracted for the categorical variable feeding habit (breastfeeding vs. bottle feeding). For other studies a narrative synthesis of the data was conducted. Publication bias was not quantitatively evaluated by Egger test or funnel plot, as there were not enough studies to be grouped in a funnel plot [38].

## Results

### Study characteristics

Seven studies were included in this systematic review (two in meta-analysis): five cross-sectional [11, 12, 16, 36, 37], one case-control [10], and one cohort [15] (Table 1). Three studies recruited children from kindergartens [12, 16, 37] and four recruited children from hospital and health centers [10, 11, 15, 36]. The age of patients ranged from 18 months to 60 months. The sample size of the studies ranged from 218 to 2395 children. Only two studies used a representative sample and both collected the sample from kindergartens, one in one of the largest cities in Syria [15] and the other in two provinces of China [12, 16].

**Table 1:** Characteristics of studies included in systematic review.

Authors (year)	Country, design	Local setting	Initial Sample (final)	Children with caries (total)	Child’s age at dental examination	Dental examination (calibration)	Feeding habit evaluation	Statistics (adjusted for confounder)	Outcomes (OR, 95% CI) or (p-value)
**Al-Dashti *et al*. (1995)**	Kuwait, cross-sectional	One hospital and one health center	227	82(179) were breastfed. 23 (30) had both feeding habits. 12(15) were bottlefed	18–48 months	2 dentists	Interview	Chi-square (no)	Breastfed children were affected by caries less frequently than bottle fed children (p<0.05); breastfed and mixed-fed (bottle+breast) children were less often affected by caries than bottle fed children (<0.05); breastfed children were less affected by caries than bottle fed and mixed fed children (p<0.01).
**Du *et al*. (2000)**	China, cross-sectional	Kindergartens in a suburban area	426	17(34) children bottle fed. 136 (392) children breast fed.	24–48 months	3 examiners (k = 0.81–0.86)	Questionnaire for the mothers	Chi-square and Logistic regression (yes)	Bottle fed children were associated with rampant caries (OR_adj_: 5.27; 95%CI: 2.16–12.89; p = 0.003). Bottle fed children associated with incisor caries (OR_adj_: 2.38; 95%CI: 1.03–4.76; p = 0.042) Bottle fed children were not associated with dental caries (OR_adj_: 0.53; 95%CI: 0.26–1.09; p = 0.08)
**Du *et al*. (2007)**	China, cross-sectional	Two provinces in China. Kindergartens in city and countryside.	2014 (1621)	59(130) bottle fed only; 604(1070) children breast fed and 218(421) both feeding habits.	36–60 months	3 examiners (k = 0.85 for interexaminer agreement)	Questionnaire for the mothers (urban) and interview (rural).	Chi-square and multivariate regression analysis: logistic and linear regression (yes)	Logistic regression: no significance between feeding habit and dental caries (p>0.05).
**Qadri *et al*. (2012)**	Syria, cross-sectional	Kindergartens	400	121(192) children were bottle fed. 71 were breastfed.	36–60 months	1 pediatric dentist (NR)	Interview with parents	Chi-square, Z statistic, Logistic regression (yes)	Breastfed children were less associated with ECC* (OR_adj_: 0.27; 95%CI: 0.18–0.41; p<0.001) and less associated with dmft† (OR:0.61; 95%CI: 0.39–0.97; p = 0.038). Higher number of teeth affected by ECC in bottle fed children (p = 0.036)
**Perera *et al*. (2014)**	Sri Lanka, cross-sectional	Pediatric Unit at the University Hospital	300 (285)	88(176) were exclusively breastfed. 48(109) were non exclusively breastfed.	36–60 months	2 medical graduates	Interview	Odds ratio and student t test (no)	The mean DEFT did not reveal a statistically significant difference between breastfed children and bottle fed children (p = 0.28). Breastfed children had a higher prevalence of caries than bottle fed children (OR = 1.27; 95% CI = 0.79–2.05).
**Roberts *et al*. (1994)**	South Africa, case-control	Health centers	109 cases 109 controls	34(75) were breastfed. 21(34) were bottlefed	12–48 months	Examiner (K = 0.95 for intra and interexaminer agreement)	Interview	Chi-square and Wilcoxon test (yes)	No statistically significant difference was found between breastfed children and bottle fed children (p>0.05).
**Majorana *et al*. (2014)**	Italy, cohort	Obstetric ward of the city hospital	2517 (2395)	‡348(588); ‖563(735); ᵜ 492 (534); ¤ 533(538)	24–30 months	2 examiners (K = 0.84 for intra examiner agreement)	Questionnaire for the mothers at birth and then with 6, 9 and 12 months, including dietary diary. One clinical examination by the age of 24–30 months.	Ordered logistic regression (yes)	Comparison between exclusively breastfed‡; moderate-high mixed fed‖, low mixed fedᵜ, exclusive artificial formula¤ and caries severity—ICDAS score. Children with a higher proportion of breast milk had a lower ICDAS score (p<0.01, log likelihood = -1956.14, OR (Standard Error) = 6.75 (0.40), 95% CI = 6.00–7.58).

OR_adj_ = Odds ratio adjusted

ECC = Early childhood caries

† Dmft = decayed tooth, decayed tooth indicated for extraction, filled tooth

‡Exclusive breast milk = 100% breast milk.

‖Moderate-High mixed feeding = 58–99% breast milk.

ᵜLow mixed feeding = 1–57% breast milk.

¤Exclusive use of formula = 0% breast milk.

All studies included assessment of feeding habits by questionnaire [15, 37], interview [10, 11, 16, 36] or both, where an interview was used for the rural population and a questionnaire for the urban population [12]. The sample of the case-control study was drawn from a main study group of 1263 children in South African communities [39]. In this study, children aged one to four years were randomly selected from the birth records of every child of the community, targeting 300 children from each geographical area. First, children with dental caries were segregated from the main sample, giving a total of 109. These were matched with 109 children without dental caries for age, gender, race and social class.

The cohort study [15] analyzed children from a hospital from birth to up to 30 months of age. Feeding habits were identified through a questionnaire applied at birth, and then again at 6, 9 and 12 months. After feeding assessment, one clinical examination was conducted by two examiners between 24 and 30 months.

### Diagnosis of dental caries

Most studies used WHO criteria[11, 12, 36, 37], ICDAS [15] or specific definition [10] for diagnosis of dental caries, while one study used three different criteria (those were ICDAS, WHO and Nyvad) [16]. One author [37] divided the presence of caries presence into three classifications: caries; rampant caries and incisor caries. The “with caries” group was defined according to WHO criteria [40], rampant caries was defined as two or more upper deciduous incisors with carious labial or palatal surfaces, while incisor caries considered only this tooth group.

### Feeding habits

All studies considered categorical data regarding the presence and absence of breastfeeding, bottle feeding or mixed feeding, although the criteria used to define types of feeding differed between studies. One author considered breast feeding or bottle feeding at birth [36]; two authors considered feeding habits up to 6 months or more [11, 15], one author considered exclusive breastfeeding up to 12 months [10], and others considered feeding habits during infancy [12, 16, 37].

### Meta-analysis

**Fig 3:**
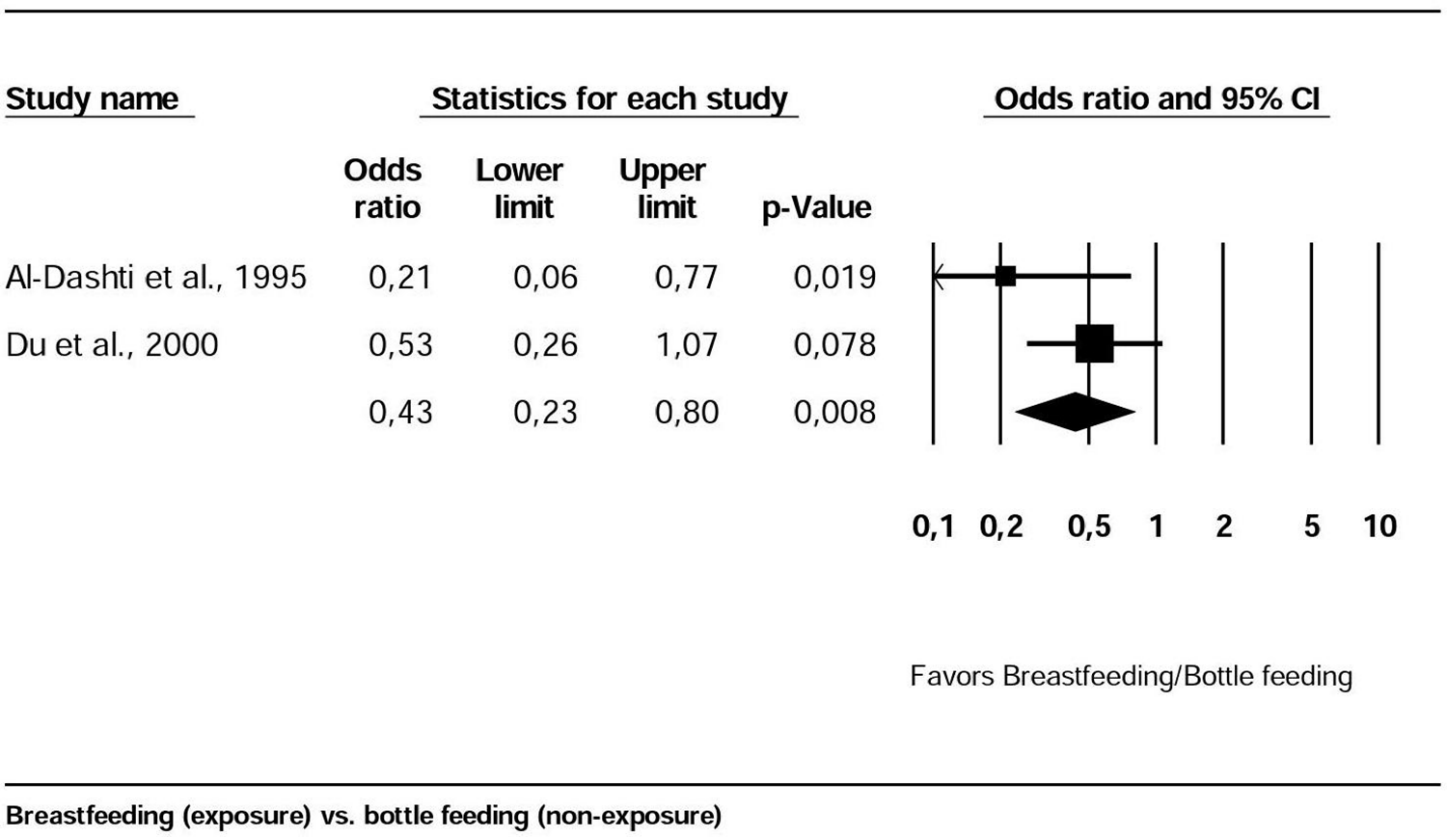
Forest plot of meta-analysis for four cross-sectional studies. Evaluates bottle or breast feeding practices and dental caries (outcome: presence of dental caries vs. absence of dental caries). Pooled effect measures [odds ratio (OR) and 95% confidence interval (CI)] indicated no statistically significant difference between breast and bottle fed children. I^2^ = 33.14%. Fixed effect model used.

Meta-analysis was initially conducted in four cross sectional studies [11, 12, 36, 37], which presented categorical variables that could be grouped (breastfeeding vs. bottle feeding). A sensitivity test was conducted and two outliers were removed [11, 12]. The final meta-analysis included two cross-sectional studies and showed that breast fed children were less affected by dental caries than bottle fed children (OR: 0.43, 95%CI: 0.23-.08, I^2^: 30.14%) (Fig 3).

### Methodological Quality Assessment

The summary of quality assessment is summarized in Fig 2. A high risk of bias was obtained when the item did not fulfill the Newcastle-Ottawa criteria, and the response given for the item was ‘no, the item has high risk of bias” [31]. Four items were judged as having a high risk of bias in a number of studies: failure to adjust for any confounding variables [11, 16, 36], representativeness [10, 11, 15, 36, 37] and ascertainment for feeding habits [15]. The confounding variables were searched for in the Methods and Results section and in the tables of the published papers.

## Discussion

### Methodological Quality Assessment

The diversity of study designs in this review was analyzed using an adapted version of the Newcastle-Ottawa scale for assessing the quality of studies. The process was made complex [41] due to the heterogeneity of studies and differences in feeding habits and dental caries classification.

While most of studies used WHO criteria [11, 12, 16, 36, 37] for diagnosing dental caries, the cohort study [15] used ICDAS criteria. Besides the diagnostic criteria, the authors of this study divided the practice of feeding into a gradative scale of exclusively breast/bottle feeding and mixed feeding.

Some studies had high risk of bias for comparability of variables. Adjustment for confounders in cross-sectional studies was performed only for social class [12, 37]. The case-control study [10] matched cases and controls for social class, age, gender and race in order to reduce confounding bias. However, the study did not adjust the confounders in a multivariate model. Adjustment was made for social class but in respect of the severity of caries in the cohort study. This study defined one of the outcomes (dependent variable) as severity of caries, as the authors used the ICDAS scale to measure severity of dental caries [15]. None analyzed bottle content during bottle feeding. As none of the studies were adjusted for all the confounding factors, all are susceptible to residual confounding. Confounding variables can include social class, hygiene and sugar in bottle content, ethnicity, early preventive dental visits, water fluoridation and on-demand feeding at night. Some of these variables such as sugar in bottle content and on-demand feeding at night can contribute to an increase in the risk of dental caries, while others can act as protective factors (water fluoridation, early preventive dental visits). These variables should be considered during data collection and should be adjusted in proper multivariate models to control the confounders.

Confounding factors have the power to mask an association or even falsely indicate an apparent association. The presence of plausible confounding makes it difficult to establish a causal link between a risk factor and outcome [42]. This makes evaluation of the role of feeding habits in the etiology of dental caries. Adjustment for major confounders such as social class, hygiene and sugar in bottle content is extremely important, as these are known to be etiological factors of dental caries [43–45].

One common reason for the decrease in quality was the absence of blindness during the ascertainment of bottle/breast feeding in relation to dental caries (exposure/outcome). Only examiners from one study [36] were unaware of the responses of mothers about feeding practices when the clinical examination for dental caries was performed. Risk of bias assessment emphasized selection bias because of inadequate or unclear allocation sequence and concealment. Lack of or unclear blinding statement can generate detection bias.

Attrition bias was low risk as all studies declared the withdrawal of participants, which did not exceed 20% (exposure/outcome). A low risk of reporting bias was observed as most of studies adequately reported outcome through a validated dental caries diagnosis index. However, observer bias may be present, as there was a lack of inter- and intra- examiner statistical measurement, such as kappa. Additionally, memory bias is inherent to the ascertainment of feeding habits, as mothers are required to report the food intake of their children. Cohort designs with real time investigation of feeding habits [15] can minimize memory bias.

Only two studies were allocated points for representativeness criteria [12, 16]. Both of these used stratified random sampling of kindergartens before randomized sampling was used to select children. The locations for sample selection were kindergartens, which were created for children whose parents worked outside the home [46]. Samples from these locations may favor specific social classes, leading to selection bias. Furthermore, many children may not be enrolled at kindergartens and can be cared for at home by a childminder or mother, leading to selecting of the sample. Moreover, there was no mention of whether these were public or private kindergartens. For this reason, the generalizability of these studies is limited.

Inter- and intra-examiner reproducibility of recordings was not evaluated in all of the studies. Studies evaluated inter-examiner agreement [12, 37]; intra-examiner agreement by Cohen’s Kappa Coefficient [15]; or both [10]. Some studies did not report any calibration testing [11] [16, 36].The lack of a kappa statistic is also a critical issue in the studies [11 16 36], as this test is considered the most reliable way to assess the agreement of researchers during data collection [47, 48]. The absence of this assessment may produce bias and produce unreliable data and in consequence, unreliable results.

Data relating to feeding habits was collected through interviews with carers or mothers of children. This type of data collection may be subject to bias due to forgetfulness or inability to provide more precise information, called information bias. All but one of the studies assessed feeding habits through questionnaires or interviews, while the remaining study [15] used a dietary diary for data collection in an attempt to reduce memory bias. However, it is important to clarify that this was only possible because it was a cohort study. A dietary diary consists of an individual writing down his or her entire food intake during a day. If this procedure is repeated regularly during a study, it could capture a more realistic view of the subject’s feeding habits.

Information bias could not be measured quantitatively due to the imprecise information regarding feeding habits given by carers. Based on their knowledge of the importance of breastfeeding, mothers may overestimate the duration of breastfeeding. For example, meta-analysis used the information from the categorical variable “breastfeeding” vs. “bottle feeding”. Three studies reported this categorical variable but did not report time data for this question [36, 37, 12]. Mothers were able to answer “yes” for breastfeeding irrespective of the duration of breastfeeding, which could vary from one month for some mothers to 6 months for others. Another study used the categorical variable “breastfeeding for up to 6 months” [11]. This study was removed in the final forest plot however, as it was an outlier. It is possible, therefore, that the clinical heterogeneity of this study influenced its statistical heterogeneity [33]. In summary, information bias regarding feeding habits must be assumed in the meta-analysis. Furthermore, psychological aspects are important in the decision of when to wean from the breast [49].

### Strength of evidence

Randomized clinical trials were not found. This was expected because of the ethical questions related to the issue. Three cross-sectional [16, 36, 37] studies and the cohort [15] study showed that breastfed children were significantly less frequently affected by caries than bottle fed children. While the cross-sectional design features a lower level of evidence and may not give a cause-and-effect relationship [46], the cohort design may indicate a temporal sequence between exposure and outcome and allow the incidence of disease to be calculated [47]. Furthermore, such studies have a higher level of evidence. Analysis of summary effect measure found these studies to be in agreement, revealing that breastfeeding had a protective effect against dental caries when compared to bottle feeding. However, some meta-analysis points should be considered: 1) the summary effect measure is drawn from cross-sectional studies, which have the lowest strength of evidence; 2) there is a risk of information bias as discussed above; 3) there was some statistical heterogeneity; 4) the number of included studies was low; 5) it was impossible to adjust for bottle content. Contrastingly, there are also some positive points as both studies included are similar and used WHO criteria to diagnose dental caries [36, 37], demonstrating clinical and methodological homogeneity.

While the majority of studies suggest the benefits of breastfeeding for dental caries, two cross-sectional [11, 12] and the case-control study [10] did not find statistical significance for this association. While case-control studies have an intermediate level of evidence, these studies, together with cohort studies, had a low risk of bias [10, 15].

Meta-analysis regarding breastfeeding duration could not be performed due to the impossibility of extracting this data. Studies showed clinical heterogeneity as the duration of breastfeeding varied from one study to another. One systematic review aimed to determine the association between duration of breastfeeding and dental caries. Children who were breastfed for longer than 12 months have fewer dental caries than those exposed to breastfeeding for a shorter time. Also, nocturnal breastfeeding longer than 12 months should not be encouraged, as it was found to increase the prevalence of dental caries in children [50]. The systematic review pooled data of breast feeding for a 12 month period only, with no other cuts off analyzed and no meta-analysis comparing breast and bottle feeding performed. The present systematic review is the first attempt to meta-analyze the association between dental caries and breastfeeding and bottle feeding practices. While both systematic reviews are different their findings are complementary.

This systematic review involved a search of multiple electronic databases, with no year of publication restriction. Efforts were made to try to find unpublished studies through grey literature. Some shortcomings of this systematic review are the presence of many Asian studies, and the exclusion of studies written in other language than English. These points can imply some publication bias, although the search of grey literature may reduce its impact [44]. These shortcomings limit the global extrapolation of these conclusions, as the concentration of Asian studies may lead to an unrepresentative sample of studies [45].

Current scientific evidence suggests that breastfeeding has a greater protective effect against dental caries than bottle feeding. This review is the first to attempt to compare the rate of dental caries rate in breastfed and bottle fed children. Breast feeding benefits the systemic health of children [6, 51] and for this reason, exclusive breastfeeding of children for at least six months is prudent [8].

## Conclusion

The available scientific evidence showed that breastfeeding is more effective at preventing dental caries in early childhood than bottle feeding. Although the duration of breastfeeding in the studies analyzed could not be determined in the present systematic review, breastfeeding should be encouraged as the exclusive feeding method for up to 6 months, followed by complementary breastfeeding for up to two years of age by all children, in accordance with WHO/UNICEF recommendations. Further prospective cohort studies with follow ups during childhood, blinding during dental examination, and control of confounders are suggested for future studies.

## Supporting Information

S1 PRISMA Checklist PRISMA 2009 Checklist. (DOC)

S1 Appendix List of all titles and abstracts for analysis and reasons for exclusion. (DOC)

S2 Appendix List of titles selected for full text analysis and the reasons for exclusion. (DOC)
