# Supplementary material for: Breast and Bottle Feeding as Risk Factors for Dental Caries: A Systematic Review and Meta-Analysis
Source: PLoS One. 2015 Nov 18;10(11):e0142922. doi: 10.1371/journal.pone.0142922 (PMC4651315; doi:10.1371/journal.pone.0142922)
Supplement: S1 Appendix — (DOC) [file pone.0142922.s002.doc]

| 1. Carrasco M, Arriagada C, Gomez S. Early childhood caries and prolonged night breast feeding. Journal of Dental Research 2003; 82:302.   **S1 Appendix. List of all titles and abstracts for analysis and reasons for exclusion.** | Wrong reference. This paper does not exist in this journal. |
| --- | --- |
| 1. Olojugba OO, Hardwick JL. Relationship of Breast-Feeding and Bottle-Feeding During Infancy to Caries Experience in Nigerian Children. Caries Research 1979; 13(2):101-102. | Text could not be found |
| 1. Abbey LM. Breast-Feeding and Caries - Reply. Journal of the American Dental Association 1979; 99(1):12 | Letter to the editor |
| 1. Campbell OA. Breast-Feeding and Caries. Journal of the American Dental Association 1979; 98(5):691-692. | Letter to the editor |
| 1. Does breastfeeding increase risk of early childhood caries? J Can Dent Assoc 2013; 79:d123. | Guideline |
| 1. Staskiewicz T. [Analysis of the influence of some factors on the intensity of early childhood caries]. Ann Acad Med Stetin 2012; 58(2):36-39. | Language other than English other than English |
| 1. Smilematters patient fact sheet. February is National Children's Dental Health Month. J Mich Dent Assoc 2011; 93(2):15. | Patient’s guide |
| 1. Zhong ZQ. [The relationship between the infant nursing bottle caries and the feeding patterns, oral health behavior and parents' oral health information]. Shanghai Kou Qiang Yi Xue 2009; 18(6):588-591.. | Language other than English other than English |
| 1. ODA patient's page. Baby bottle syndrome. J Okla Dent Assoc 2009; 100(9):7. | Patient’s guide |
| 1. Ribeiro NM, Ribeiro MA. Breastfeeding and early childhood caries: a myth that survives. J Pediatr (Rio J ) 2009; 85(5):464-465. | Letter to the editor |
| 1. Jigjid B, Ueno M, Shinada K, Kawaguchi Y. Early childhood caries and related risk factors in Mongolian children. Community Dent Health 2009; 26(2):121-128. | Does not compare breastfeeding and bottlefeeding |
| 1. Khamadeeva AM, Demina RR, Bagdasarova OA, Nogina n. [Role of behaviorial risk factors in developing dental caries of temporary teeth in infancy]. Stomatologiia (Mosk) 2008; 87(5):68-71. | Language other than English |
| 1. Menghini G, Steiner M, Thomet E, Roos M, Imfeld T. Caries prevalence in 2-year-old children in the city of Zurich. Community Dent Health 2008; 25(3):154-160. | Does not compare breastfeeding and bottlefeeding |
| 1. Menghini G, Steiner M, Imfeld T. [Early childhood caries--facts and prevention]. Ther Umsch 2008; 65(2):75-82. | Language other than English |
| 1. van Palenstein Helderman WH, Holmgren CJ. Research into factors which might contribute to the prevalence and severity of dental caries. Caries Res 2008; 42(2):155-156. | Letter to the editor |
| 1. Faye M, Ba AA, Yam AA, Ba I. [Caries patterns and diet in early childhood caries]. Dakar Med 2006; 51(2):72-77. | Language other than English other than English |
| 1. Abdoll GS. Report on the nursing bottle caries campaign launched by the Free State Oral Health Services. SADJ 2001; 56(1):32-33. | Different outcome, not dental caries |
| 1. Baginska J, Stokowska W. [Dietary habits and early childhood caries intensity among young children]. Wiad Lek 2006; 59(1-2):5-9. | Language other than English other than English |
| 1. Bringing up baby's teeth. CDS Rev 2005; 98(7):29. | Patient educational hand out |
| 1. Policy on dietary recommendations for infants, children, and adolescents. Pediatr Dent 2005; 27(7 Suppl):36-37. | Guideline |
| 1. Policy on early childhood caries (ECC): classifications, consequences, and preventive strategies. Pediatr Dent 2005; 27(7 Suppl):31-33. | Guideline |
| 1. Ge XJ, Zhang BS, Li B, Zhao LJ, Zhao B, Ren XY et al. [The effects of feeding methods on deciduous caries.]. Shanghai Kou Qiang Yi Xue 2004; 13(5):365-366. | Language other than English other than English |
| 1. Ye W, Feng XP, Liu YL. [An epidemiological study of risk factors of rampant caries in Shanghai children]. Shanghai Kou Qiang Yi Xue 2001; 10(2):166-169. | Language other than English other than English |
| 1. Mizoguchi K, Kurumado K, Tango T, Minowa M. [Study on factors for caries and infant feeding characteristics in children aged 1.5-3 years in a Kanto urban area]. Nihon Koshu Eisei Zasshi 2003; 50(9):867-878. | Language other than English other than English |
| 1. Ramezani GH, Norozi A, Valael N. The prevalence of nursing caries in 18 to 60 months old children in Qazvin. J Indian Soc Pedod Prev Dent 2003; 21(1):19-26. | Does not compare breastfeeding and bottlefeeding |
| 1. Wyne AH, Chohan AN, al-Begomi R. Feeding and dietary practices of nursing caries children in Riyadh, Saudi Arabia. Odontostomatol Trop 2002; 25(100):37-42. | Prevalence feeding habits |
| 1. Hallett KB, O'Rourke PK. Early childhood caries and infant feeding practice. Community Dent Health 2002; 19(4):237-242. | Does not compare breastfeeding and bottlefeeding |
| 1. Sayegh A, Dini EL, Holt RD, Bedi R. Caries prevalence and patterns and their relationship to social class, infant feeding and oral hygiene in 4-5-year-old children in Amman, Jordan. Community Dent Health 2002; 19(3):144-151. | Does not compare breastfeeding and bottlefeeding |
| 1. Acs G, Ng MW. Early childhood caries and well being. Pediatr Dent 2002; 24(4):288. | Letter to the editor |
| 1. Greer MH, Tendan SL. Early childhood dental caries in Hawai'i. Hawaii Dent J 1998; 29(2):10, 14. | Title not interest |
| 1. Erickson PR, Nickman JD. Early childhood caries: etiology, risk assessment, and prevention. Northwest Dent 1999; 78(6):27-32. | Review |
| 1. Hallett KB. Early childhood caries--a new name for an old problem. Ann R Australas Coll Dent Surg 2000; 15:268-275. | Title not interest |
| 1. Creedon MI, O'Mullane DM. Factors affecting caries levels amongst 5-year-old children in County Kerry, Ireland. Community Dent Health 2001; 18(2):72-78. | Does not compare breastfeeding and bottlefeeding |
| 1. Davies GM, Blinkhorn FA, Duxbury JT. Caries among 3-year-olds in greater Manchester. Br Dent J 2001; 190(7):381-384. | Prevalence |
| 1. Behrendt A, Sziegoleit F, Muler-Lessmann V, Ipek-Ozdemir G, Wetzel WE. Nursing-bottle syndrome caused by prolonged drinking from vessels with bill-shaped extensions. ASDC J Dent Child 2001; 68(1):47-50, 12. | Different ethiology |
| 1. Usatine R. Severe caries in a child. West J Med 2001; 174(3):167-168. | Case report |
| 1. Peretz B, Eidelman E. ["Baby bottle tooth decay"--a risk to the teeth of babies and young infants]. Harefuah 1998; 134(9):731-734. | Language other than English |
| 1. Ye W, Feng XP, Liu YL. Epidemiological study of the risk factors of rampant caries in Shanghai children. Chin J Dent Res 1999; 2(2):58-62. | Does not compare breastfeeding and bottlefeeding |
| 1. Ramos-Gomez FJ, Tomar SL, Ellison J, Artiga N, Sintes J, Vicuna G. Assessment of early childhood caries and dietary habits in a population of migrant Hispanic children in Stockton, California. ASDC J Dent Child 1999; 66(6):395-403, 366. | Does not compare breasfeeding and bottlefeeding |
| 1. Sinton J, Valaitis R, Passarelli C, Sheehan D, Hesch R. A systematic overview of the relationship between infant feeding caries and breast-feeding. Ont Dent 1998; 75(9):23-27. | Review |
| 1. Quartey JB, Williamson DD. Prevalence of early childhood caries at Harris County clinics. ASDC J Dent Child 1999; 66(2):127-31, 85. | Prevalence |
| 1. Erickson PR, Mazhari E. Investigation of the role of human breast milk in caries development. Pediatr Dent 1999; 21(2):86-90. | Different ethiology |
| 1. Smith PJ, Moffatt ME. Baby-bottle tooth decay: are we on the right track? Int J Circumpolar Health 1998; 57 Suppl 1:155-162. | Review |
| 1. Ollila P, Niemela M, Uhari M, Larmas M. Prolonged pacifier-sucking and use of a nursing bottle at night: possible risk factors for dental caries in children. Acta Odontol Scand 1998; 56(4):233-237. | Does not compare breastfeeding and bottlefeeding |
| 1. Maupome G. An introspective qualitative report on dietary patterns and elevated levels of dental decay in a deprived urban population in northern Mexico. ASDC J Dent Child 1998; 65(4):276-85, 230. | Prevalence |
| 1. Lopez D, V, Velazquez-Quintana Y, Weinstein P, Domoto P, Leroux B. Early childhood caries and risk factors in rural Puerto Rican children. ASDC J Dent Child 1998; 65(2):132-135. | Does not compare breastfeeding and bottlefeeding |
| 1. Weerheijm KL, Uyttendaele-Speybrouck BF, Euwe HC, Groen HJ. Prolonged demand breast-feeding and nursing caries. Caries Res 1998; 32(1):46-50. | Does not compare breastfeeding and bottlefeeding |
| 1. Harrison R, Wong T, Ewan C, Contreras B, Phung Y. Feeding practices and dental caries in an urban Canadian population of Vietnamese preschool children. ASDC J Dent Child 1997; 64(2):112-117. | Does not compare breastfeeding and bottlefeeding |
| 1. Henry RJ. Why do 20% of our children experience 80% of the decay? An update on the status of childhood caries. Tex Dent J 1997; 114(1):10-14. | Review |
| 1. Febres C, Echeverri EA, Keene HJ. Parental awareness, habits, and social factors and their relationship to baby bottle tooth decay. Pediatr Dent 1997; 19(1):22-27. | Does not compare breastfeeding and bottlefeeding |
| 1. Holt RD, Winter GB, Downer MC, Bellis WJ, Hay IS. Caries in pre-school children in Camden 1993/94. Br Dent J 1996; 181(11-12):405-410. | Prevalence |
| 1. Bernshaw N. Prolonged breastfeeding and dental caries. J Hum Lact 1996; 12(4):277. | Letter to the editor |
| 1. Moynihan PJ, Holt RD. The national diet and nutrition survey of 1.5 to 4.5 year old children: summary of the findings of the dental survey. Br Dent J 1996; 181(9):328-332. | Prevalence |
| 1. Weinstein P, Smith WF, Fraser-Lee N, Shimono T, Tsubouchi J. Epidemiologic study of 19-month-old Edmonton, Alberta children: caries rates and risk factors. ASDC J Dent Child 1996; 63(6):426-433. | Does not compare breastfeeding and bottlefeedng |
| 1. Truhe T. Diet and caries. Dent Today 1996; 15(9):60, 62-60, 65. | Review |
| 1. Ayhan H. Influencing factors of nursing caries. J Clin Pediatr Dent 1996; 20(4):313-316. | Title not interest |
| 1. Alaluusua S, Matto J, Gronroos L, Innila S, Torkko H, Asikainen S et al. Oral colonization by more than one clonal type of mutans streptococcus in children with nursing-bottle dental caries. Arch Oral Biol 1996; 41(2):167-173. | In vitro study |
| 1. Muller M. Nursing-bottle syndrome: risk factors. ASDC J Dent Child 1996; 63(1):42-50. | Letter to the editor |
| 1. Wendt LK, Birkhed D. Dietary habits related to caries development and immigrant status in infants and toddlers living in Sweden. Acta Odontol Scand 1995; 53(6):339-344. | Prevalence |
| 1. Von Burg MM, Sanders BJ, Weddell JA. Baby bottle tooth decay: a concern for all mothers. Pediatr Nurs 1995; 21(6):515-9, quiz. | Patient’s guide |
| 1. Tinanoff N. Dental caries risk assessment and prevention. Dent Clin North Am 1995; 39(4):709-719. | Review |
| 1. Tsubouchi J, Tsubouchi M, Maynard RJ, Domoto PK, Weinstein P. A study of dental caries and risk factors among Native American infants. ASDC J Dent Child 1995; 62(4):283-287. | Prevalence |
| 1. Duperon DF. Early childhood caries: a continuing dilemma. J Calif Dent Assoc 1995; 23(2):15-2. | Review |
| 1. Matee M, van't Hof M, Maselle S, Mikx F, van Palenstein HW. Nursing caries, linear hypoplasia, and nursing and weaning habits in Tanzanian infants. Community Dent Oral Epidemiol 1994; 22(5 Pt 1):289-293. | Different etiology: hypoplasia |
| 1. Alaluusua S, Malmivirta R. Early plaque accumulation--a sign for caries risk in young children. Community Dent Oral Epidemiol 1994; 22(5 Pt 1):273-276. | Prevalence and other etiology: plaque accumulation |
| 1. Todd RV, Durward CS, Chot C, So PK, Im P. The dental caries experience, oral hygiene and dietary practices of preschool children of factory workers in Phnom Penh, Cambodia. Int J Paediatr Dent 1994; 4(3):173-178. | Prevalence |
| 1. Tsubouchi J, Higashi T, Shimono T, Domoto PK, Weinstein P. A study of baby bottle tooth decay and risk factors for 18-month old infants in rural Japan. ASDC J Dent Child 1994; 61(4):293-298. | Does not compare breastfeeding and bottlefeeding |
| 1. Degano MP, Degano RA. Breastfeeding and oral health. A primer for the dental practitioner. N Y State Dent J 1993; 59(2):30-32. | Review |
| 1. Leverett DH, Featherstone JD, Proskin HM, Adair SM, Eisenberg AD, Mundorff-Shrestha SA et al. Caries risk assessment by a cross-sectional discrimination model. J Dent Res 1993; 72(2):529-537. | Different etiology: fluoridated water |
| 1. Schwartz SS, Rosivack RG, Michelotti P. A child's sleeping habit as a cause of nursing caries. ASDC J Dent Child 1993; 60(1):22-25. | Sleeping habit |
| 1. O'Sullivan DM, Tinanoff N. Social and biological factors contributing to caries of the maxillary anterior teeth. Pediatr Dent 1993; 15(1):41-44. | Does not compare breastfeeding and bottle feeding |
| 1. Schulte JR, Druyan ME, Hagen JC. Early childhood tooth decay. Pediatric interventions. Clin Pediatr (Phila) 1992; 31(12):727-730. | Different etiology |
| 1. Henderson HZ. Baby bottle tooth decay. Indiana Med 1992; 85(3):197. | Letter to the editor |
| 1. Kovesi T, Levison H. The "companion bottle": a useful predictor of children at risk for the development of nursing bottle caries. Pediatrics 1992; 89(5 Pt 1):976-977. | Letter to the editor |
| 1. Silver DH. A comparison of 3-year-olds' caries experience in 1973, 1981 and 1989 in a Hertfordshire town, related to family behaviour and social class. Br Dent J 1992; 172(5):191-197. | Prevalence |
| 1. Matee MI, Mikx FH, Maselle SY, van Palenstein Helderman WH. Mutans streptococci and lactobacilli in breast-fed children with rampant caries. Caries Res 1992; 26(3):183-187. | In vitro study |
| 1. Eronat N, Eden E. A comparative study of some influencing factors of rampant or nursing caries in preschool children. J Clin Pediatr Dent 1992; 16(4):275-279. | Does not compare breastfeeding and bottlefeeding |
| 1. Hara AT, Kelly SA, Gonzalez-Cabezas C, Eckert GJ, Barlow AP, Mason SC et al. Influence of fluoride availability of dentifrices on eroded enamel remineralization in situ. Caries Res 2009; 43(1):57-63. | in vitro study |
| 1. Mangskau K. Baby bottle tooth decay: a problem affecting young children in North Dakota. Northwest Dent 1991; 70(6):25. | Letter to the editor |
| 1. O'Malley B, Brown AC, Tate M, Hertzler AA, Rojas MH. Infant feeding practices of migrant farm laborers in northern Colorado. J Am Diet Assoc 1991; 91(9):1084-1087. | Different outcome and prevalence |
| 1. Aaltonen AS. The frequency of mother-infant salivary close contacts and maternal caries activity affect caries occurrence in 4-year-old children. Proc Finn Dent Soc 1991; 87(3):373-382. | Different etiology |
| 1. Houde G, Gagnon PF, St-Germain M. A descriptive study of early caries and oral health habits of Inuit pre-schoolers: preliminary results. Arctic Med Res 1991; Suppl:683-684. | Descriptive study. |
| 1. Alaluusua S, Myllarniemi S, Kallio M, Salmenpera L, Tainio VM. Prevalence of caries and salivary levels of mutans streptococci in 5-year-old children in relation to duration of breast feeding. Scand J Dent Res 1990; 98(3):193-196. | In vitro study |
| 1. Kurth A. [Nursing bottle caries]. Rev Dent Chile 1990; 81(1):10-14. | Language other than English other than English |
| 1. Todd R, Gelbier S. Dental caries prevalence in Vietnamese children and teenagers in three London boroughs. Br Dent J 1990; 168(1):24-26. | Prevalence |
| 1. Liu J. Neglected problem: nursing bottle syndrome. Dentistry (Loma Linda ) 1990; 3(2):57-58. | Case report |
| 1. Buhl S, Wetzel WE, Bodeker RH. [Studies on the incidence of caries in 6- to 48-month old infants]. Dtsch Zahnarztl Z 1989; 44(9):673-677. | Language other than English other than English |
| 1. Marino RV, Bomze K, Scholl TO, Anhalt H. Nursing bottle caries: characteristics of children at risk. Clin Pediatr (Phila) 1989; 28(3):129-131. | Does not compare breastfeeding and bottlefeeding |
| 1. Amaratunge A. Rampant dental caries in Papua New Guinean children. Odontostomatol Trop 1989; 12(1):14-16. | Prevalence |
| 1. Wetzel WE. [Nursing bottle syndrome in small children]. Zahnarztl Mitt 1989; 79(3):249-257. | Language other than English other than English |
| 1. Srkoc O, Bajan M, Stilinovic D. [Etiology of nursing caries]. Acta Stomatol Croat 1989; 23(2):159-165. | Language other than English other than English |
| 1. Johnsen DC. Baby bottle tooth decay: a preventable health problem in infants. Update Pediatr Dent 1988; 2(1):1-7. | Review |
| 1. Wetzel WE. ["Nursing bottle syndrome" in young children. Dental findings, incidence and family environment]. Monatsschr Kinderheilkd 1988; 136(10):673-679. | Language other than English other than English |
| 1. Albert RJ, Cantin RY, Cross HG, Castaldi CR. Nursing caries in the Inuit children of the Keewatin. J Can Dent Assoc 1988; 54(10):751-758. | Prevention |
| 1. Sbordone L, Di ML, Ciaglia RN, Pettoello MM, Lenci F, Di CA et al. [Dental caries in childhood. A cross-sectional epidemiologic study and correlation with a breast-feeding program]. Minerva Stomatol 1988; 37(8):655-657. | Language other than English other than English |
| 1. Jackson-Herrerias G, Angeles-Mendoza A. [Conditioning and determining factors in the incidence of the "baby-bottle syndrome"]. Bol Med Hosp Infant Mex 1988; 45(4):240-244. | Language other than English other than English |
| 1. Borresen HC. [Night breast feeding and dental health]. Tidsskr Nor Laegeforen 1987; 107(9):869-870. | Language other than English other than English |
| 1. Stabouli. [Caries and breast feeding]. Arch Odonto Estomatol 1986; 2 Spec No:86-87. | Language other than English other than English |
| 1. Aldred MJ, Wade WG, Llewelyn DR, Walker DM. Class-specific antibodies to Streptococcus mutans in human serum, saliva and breast milk. J Immunol Methods 1986; 87(1):103-108. | In vitro study |
| 1. Roeters FJ, Burgersdijk RC, Mikx FH. [Caries as a result of frequent and prolonged breast feeding]. Ned Tijdschr Tandheelkd 1986; 93(1):14-16 | Language other than English other than English |
| 1. Haq ME, Begum K, Muttalib MA, Shahidullah M. Prevalence of caries in urban children and its relation to feeding pattern. Bangladesh Med Res Counc Bull 1985; 11(2):55-63. | Prevalence |
| 1. Salako NO. Infant feeding profile and dental caries status of urban Nigerian children. Acta Odontol Pediatr 1985; 6(1):13-17. | Case report |
| 1. Gupta KS. Infant bottle feeding and dental caries among small children in Dar es Salaam-Tanzania. Odontostomatol Trop 1985; 8(2):111-112. | Case report |
| 1. Scheer B. Caries in children--the dietary factor. Middle East Dent Oral Health 1985;(3):20-22. | Letter to the editor |
| 1. Milnes AR, Bowden GH. The microflora associated with developing lesions of nursing caries. Caries Res 1985; 19(4):289-297. | In vitro study |
| 1. Janicha J, Szpringer-Nodzak M. [Natural feeding and caries in the deciduous teeth of children up to 3 years of age]. Czas Stomatol 1984; 37(9):629-631. | Language other than English other than English |
| 1. Amaratunge A, Ekanayake SL. Rampant caries in Sri Lankan children. A pilot study. Odontostomatol Trop 1984; 7(3):133-138. | Prevalence |
| 1. Gagnon PF. [Dietary habits in early childhood and the appearance of rampant caries]. J Dent Que 1984; 21:119-122. | Language other than English other than English |
| 1. Hackett AF, Rugg-Gunn AJ, Murray JJ, Roberts GJ. Can breast feeding cause dental caries? Hum Nutr Appl Nutr 1984; 38(1):23-28. | Review |
| 1. Ishikawa M, Watanabe K, Kimura K, Kikuchi J. [Relation between daily habits and caries incidence in the dental health examination of 1 1/2-year-old infants. A comparison with a survey taken 7 years previously]. Shoni Shikagaku Zasshi 1984; 22(4):846-853. | Language other than English other than English |
| 1. Arai M. [Dental caries prevalence in deciduous teeth surveyed by feeding methods and histological observations]. Tsurumi Shigaku 1984; 10(1):229-258. | Language other than English other than English |
| 1. Johnsen DC. Dental caries patterns in preschool children. Dent Clin North Am 1984; 28(1):3-20. | Case report |
| 1. Jelliffe DB, Jelliffe EF. Breast feeding could be a cause of the type of dental caries. J Dent 1983; 11(4):361. | Letter to the editor |
| 1. Brams M, Maloney J. "Nursing bottle caries" in breast-fed children. J Pediatr 1983; 103(3):415-416. | Case report |
| 1. Weyers H. [Findings in "nursing-bottle caries"]. Dtsch Zahnarztl Z 1983; 38(7):722-726. | Language other than English other than English |
| 1. Hovels O. ["Sugar-induced caries" due to excessive enjoyment of instant tea from nursing bottles]. Monatsschr Kinderheilkd 1983; 131(3):174-175. | Language other than English other than English |
| 1. Richardson BD, Cleaton-Jones PE. Infant feeding practices and nursing bottle caries. ASDC J Dent Child 1983; 50(1):72. | Letter to the editor |
| 1. Roberts GJ. Is breast feeding a possible cause of dental caries? J Dent 1982; 10(4):346-352. | Just breastfeeding |
| 1. Wetzel WE. [Dental caries caused by excessive consumption of sweetened tea from nursing bottles]. Monatsschr Kinderheilkd 1982; 130(9):726-730. | Language other than English other than English |
| 1. Johnsen DC. Characteristics and backgrounds of children with "nursing caries". Pediatr Dent 1982; 4(3):218-224. | Just bottlefeeding |
| 1. Holt RD, Joels D, Winter GB. Caries in pre-school children. The Camden study. Br Dent J 1982; 153(3):107-109. | Hygiene habits |
| 1. Derkson GD, Ponti P. Nursing bottle syndrome; prevalence and etiology in a non-fluoridated city. J Can Dent Assoc 1982; 48(6):389-393. | Different etiology |
| 1. Schouker M. [The nursing bottle syndrome]. Actual Odontostomatol (Paris) 1982; 36(140):577-586. | Language other than English other than English |
| 1. Allen KR, Leppard PI. Bottle caries - an increasing problem. Aust Dent J 1981; 26(6):403-404. | Letter to the editor |
| 1. Richardson BD, Cleaton-Jones PE, McInnes PM, Rantsho JM. Infant feeding practices and nursing bottle caries. ASDC J Dent Child 1981; 48(6):423-429. | Etiology vitamins |
| 1. Nagasawa S, Kishimoto E, Nara Y, Hatada N, Morita E, Kawano K et al. [The relationship between dental caries in 3-year-old children and feeding methods, between-meal eating habits, use of feeding bottles, and drinking of acidophilus beverages]. Koku Eisei Gakkai Zasshi 1981; 30(5):423-431.. | Language other than English other than English |
| 1. Marechaux SC, Monnier T, Arnold C. [Nursing bottle caries]. Prev Stomatol 1981; 7(1):41-46. | Language other than English other than English |
| 1. Walton JL, Messer LB. Dental caries and fluorosis in breast-fed and bottle-fed children. Caries Res 1981; 15(2):124-137. | Etiology fluorosis |
| 1. Marechaux SC, Monnier T, Arnold C. ["Baby-bottle" caries]. SSO Schweiz Monatsschr Zahnheilkd 1980; 90(11):1049-1055. | Language other than English other than English |
| 1. Cudzinowski L. ["Baby-bottle" syndrome]. Union Med Can 1980; 109(6):853-855. | Language other than English other than English |
| 1. Toors FA. [Dental caries in the first years of life]. Ned Tijdschr Geneeskd 1980; 124(1):12-16. | Language other than English other than English |
| 1. Abbey LM. Night breast feeding and dental caries. Pediatrics 1979; 64(5):701. | Letter to the editor |
| 1. Snaer WR. Breast-feeding and caries. J Am Dent Assoc 1979; 99(1):11-12. | Letter to the editor |
| 1. Snaer WR. Breast-feeding and caries. J Am Dent Assoc 1979; 98(5):691-692. | Letter to the editor |
| 1. Abbey LM. Is breast feeding a likely cause of dental caries in young children? J Am Dent Assoc 1979; 98(1):21-23. | One author. Case report |
| 1. Shearer TR, Howard HE, DeSart DJ. Breast-feeding and nursing caries. J Oreg Dent Assoc 1978; 47(3):17. | Not specified |
| 1. Jasmin JR. [Nursing bottle caries]. Pedod Fr 1978; 12:203-209. | Language other than English other than English |
| 1. Richardson BD, Cleaton-Jones PE. Nursing bottle caries. Pediatrics 1977; 60(5):748-749. | Prevalence |
| 1. Countryman BA. Kotlow and Gardner et al: recommendation on breastfeeding. ASDC J Dent Child 1977; 44(6):498. | Letter to the editor |
| 1. Shelton PG, Berkowitz RJ, Forrester DJ. Nursing bottle caries. Pediatrics 1977; 59(5):777-778. | Prevalence |
| 1. Kotlow LA. Breast feeding: a cause of dental caries in children. ASDC J Dent Child 1977; 44(3):192-193. | Case report |
| 1. Trippie RL, Jennings RE. Nursing bottle syndrome. Tex Med 1977; 73(3):47-51. | Case report |
| 1. Powell D. Milk...is it related to rampant caries of the early primary dentition? J Calif Dent Assoc 1976; 4(1):58-63. | Other etiology: milk |
| 1. Forsman B, Ericsson Y. Breastfeeding, formula feeding and dental health in low-fluoride districts in Sweden. Community Dent Oral Epidemiol 1974; 2(1):1-6. | Other etiology: fluorosis |
| 1. Miller JB. Rampant caries as a result of latent bottle feeding in young children. J Okla State Dent Assoc 1973; 63(4):9-11. | Nutrition |
| 1. Stone JH, Kroll RG. Elimination of rampamt caries due to bottle feeding. J N J Dent Assoc 1973; 44(2):39. | Just bottlefeeding. Other etiology. |
| 1. Castano FA. Night-bottle syndrome. Pa Dent J (Harrisb ) 1972; 39(1):8-11. | Not humans |
| 1. Picton DC, Wiltshear PJ. A comparison of the effects of early feeding habits on the caries prevalence of deciduous teeth. Dent Pract Dent Rec 1970; 20(5):170-172. | Other etiology: just breastfeeding |
| 1. Kroll RG, Stone JH. Nocturnal bottle-feeding as a contributory cause of rampant dental caries in the infant and young child. J Dent Child 1967; 34(6):454-459. | Other etiology: just bottle feeding |
| 1. TANK G. RECENT ADVANCES IN NUTRITION AND DENTAL CARIES. J Am Diet Assoc 1965; 46:293-297.. | Review |
| 1. NOVAK S, MECIR M, MRKLAS L. [DENTAL CARIES IN CHILDREN WHO WERE BREAST-FED AND THOSE WHO WERE ARTIFICIALLY FED DURING THE 1ST MONTH OF THEIR LIFE]. Cesk Stomatol 1965; 65:91-97. | Language other than English other than English |
| 1. TANK G, STORVICK CA. CARIES EXPERIENCE OF CHILDREN ONE TO SIX YEARS OLD IN TWO OREGON COMMUNITIES (CORVALLIS AND ALBANY). 3. RELATION OF DIET TO VARIATION OF DENTAL CARIES. J Am Dent Assoc 1965; 70:394-403. | Other etiology: just breatfeeding |
| 1. Arora A, Foster JP, Gillies D, Moxey AJ, Moody G, Curtis B. Breastfeeding for oral health in preschool children. Cochrane Database of Systematic | Review protocol |
| 1. Fluoride supplementation of the breast-fed infant. JAMA 1990; 263(16):2179. | Different etiology |
| 1. The public side of DSSNY. N Y State Dent J 1997; 63(8):44-45. | Title not interest |
| 1. Nutrition for healthy term infants. Paediatr Child Health 1998; 3(2):109-116. | Temas |
| 1. Prolonged use of sippy cups under scrutiny. Dent Today 2002; 21(5):44. | Different outcome |
| 1. For the dental patient. From baby bottle to cup. Choose training cups carefully, use them temporarily. J Am Dent Assoc 2004; 135(3):387. | Patient educational hand out |
| 1. Bringing up baby's teeth. CDS Rev 2005; 98(7):29. | Patient educational hand out |
| 1. Aaltonen AS, Tenovuo J, Lehtonen OP, Saksala R, Meurman O. Serum antibodies against oral Streptococcus mutans in young children in relation to dental caries and maternal close-contacts. Arch Oral Biol 1985; 30(4):331-335. | Title not interest |
| 1. Aaltonen AS, Tenovuo J. Association between mother-infant salivary contacts and caries resistance in children: a cohort study. Pediatr Dent 1994; 16(2):110-116. | Title not interest |
| 1. Abbey LM. Night breast feeding and dental caries. Pediatrics 1979; 64(5):701. | Letter |
| 1. ODA patient's page. Baby bottle syndrome. J Okla Dent Assoc 2009; 100(9):7. | Patient’s educational hand out |
| 1. Oral health during pregnancy and early childhood: evidence-based guidelines for health professionals. J Calif Dent Assoc 2010; 38(6):391-40. | Guideline |
| 1. Smilematters patient fact sheet. February is National Children's Dental Health Month. J Mich Dent Assoc 2011; 93(2):15. | Patient’s educational hand out |
| 1. Acs G, Lodolini G, Kaminsky S, Cisneros GJ. Effect of nursing caries on body weight in a pediatric population. Pediatr Dent 1992; 14(5):302-305. | Different outcome |
| 1. Patient's page. Keeping your child's teeth healthy begins early. J Okla Dent Assoc 2013; 104(7):8. | Patient’s educational hand out |
| 1. Acs G, Lodolini G, Shulman R, Chussid S. The effect of dental rehabilitation on the body weight of children with failure to thrive: case reports. Compend Contin Educ Dent 1998; 19(2):164-1. | Case report |
| 1. Acs G, Ng MW. Early childhood caries and well being. Pediatr Dent 2002; 24(4):288. | Letter |
| 1. Adair SM, Durr DP. The treatment of nursing bottle caries. ASDC J Dent Child 1983; 50(2):96, 154. | Letter |
| 1. Adams MN. Toothless children. S Afr Med J 1998; 88(4):469. | Letter |
| 1. Agostoni C, Baselli L, Mazzoni MB. Early nutrition patterns and diseases of adulthood: a plausible link? Eur J Intern Med 2013; 24(1):5-10. | Title not interest |
| 1. Aguirrezabal IJ. [Nursing-bottle syndrome. Apropos of a case]. An Esp Pediatr 1988; 29(2):169-171. | Case report |
| 1. Al-Jewair TS, Leake JL. The prevalence and risks of early childhood caries (ECC) in Toronto, Canada. J Contemp Dent Pract 2010; 11(5):001-008. | Different outcome |
| 1. Al-Malik MI, Holt RD, Bedi R. The relationship between erosion, caries and rampant caries and dietary habits in preschool children in Saudi Arabia. Int J Paediatr Dent 2001; 11(6):430-439. | Different outcome |
| 1. Al-Malik MI, Holt RD, Bedi R. Prevalence and patterns of caries, rampant caries, and oral health in two- to five-year-old children in Saudi Arabia. J Dent Child (Chic ) 2003; 70(3):235-242. | Different etiology |
| 1. Alaluusua S, Gronroos L, Zhu X, Saarela M, Matto J, Asikainen S et al. Production of glucosyltransferases by clinical mutans streptococcal isolates as determined by semiquantitative cross-dot assay. Arch Oral Biol 1997; 42(6):417-422. | Title not interest |
| 1. Albert DA, Park K, Findley S, Mitchell DA, McManus JM. Dental caries among disadvantaged 3- to 4-year-old children in northern Manhattan. Pediatr Dent 2002; 24(3):229-233. | Different outcome |
| 1. Allen KR, Leppard PI. Bottle caries - an increasing problem. Aust Dent J 1981; 26(6):403-404. | Letter |
| 1. Alley JW, Alderman EJ, Levy SM, Kuthy RA. Dietary fluoride supplements for children--the role of the physician. J Med Assoc Ga 1989; 78(9):629-632. | Different outcome |
| 1. Allison PJ, Schwartz S. Interproximal contact points and proximal caries in posterior primary teeth. Pediatr Dent 2003; 25(4):334-340. | Title not interest |
| 1. Ankola AV, Nagesh L, Hegde P, Karibasappa GN. Primary dentition status and treatment needs of children with cleft lip and/or palate. J Indian Soc Pedod Prev Dent 2005; 23(2):80-82. | Different etiology |
| 1. Arora A, McNab MA, Lewis MW, Hilton G, Blinkhorn AS, Schwarz E. 'I can't relate it to teeth': a qualitative approach to evaluate oral health education materials for preschool children in New South Wales, Australia. Int J Paediatr Dent 2012; 22(4):302-309. | Title not interest |
| 1. Askarizadeh N, Siyonat P. The prevalence and pattern of nursing caries in preschool children of Tehran. J Indian Soc Pedod Prev Dent 2004; 22(3):92-95. | Different etiology |
| 1. Ayhan H, Suskan E, Yildirim S. The effect of nursing or rampant caries on height, body weight and head circumference. J Clin Pediatr Dent 1996; 20(3):209-212. | Different outcome |
| 1. Azarpazhooh A, Limeback H, Lawrence HP, Shah PS. Xylitol for preventing acute otitis media in children up to 12 years of age. Cochrane Database of Systematic Reviews 2011. | Title not interest |
| 1. Baghianimoghadam MH, Nadrian H, Rahaei Z. The Effects of Education on Formula and Bottle-Feeding Behaviors of Nursing Mothers Based on PRECEDE Model. Iranian Journal of Pediatrics 2009; 19(4):359-366. | Title not interest |
| 1. Bakalian S, Lewis CW. Question from the clinician: fluoridated water. Pediatr Rev 2003; 24(2):70. | Title not interest |
| 1. Baker KA, Levy SM. Review of systemic fluoride supplementation and consideration of the pharmacist's role. Drug Intell Clin Pharm 1986; 20(12):935-942. | Title not interest |
| 1. Barnes GP, Parker WA, Lyon TC, Jr., Drum MA, Coleman GC. Ethnicity, location, age, and fluoridation factors in baby bottle tooth decay and caries prevalence of Head Start children. Public Health Rep 1992; 107(2):167-173. | Different etiology |
| 1. Barras E. [Bottle fed children with caries: dental care under general anesthesia]. Rev Med Suisse Romande 1998; 118(1):7-12. | Language other than English other than English |
| 1. Baum CR, Shannon MW. Lead and caries. Nat Med 1998; 4(1):3. | Letter |
| 1. Bellis MA, Jarman I, Downing J, Perkins C, Beynon C, Hughes K et al. Using clustering techniques to identify localities with multiple health and social needs. Health Place 2012; 18(2):138-143. | Title not interest |
| 1. Benitez C, O'Sullivan D, Tinanoff N. Effect of a preventive approach for the treatment of nursing bottle caries. ASDC J Dent Child 1994; 61(1):46-49. | Title not interest |
| 1. Berkenboom M. [Multi-caries in the young child]. Rev Belge Med Dent (1984 ) 1992; 47(1):75-82. | Case report |
| 1. Berkowitz R. Etiology of nursing caries: a microbiologic perspective. J Public Health Dent 1996; 56(1):51-54. | Review |
| 1. Berkowitz RJ, Turner J, Hughes C. Microbial characteristics of the human dental caries associated with prolonged bottle-feeding. Arch Oral Biol 1984; 29(11):949-951. | Different etiology |
| 1. Berkowitz RJ. Streptococcus mutans and dental caries in infants. Compend Contin Educ Dent 1985; 6(6):463-466. | Different etiology |
| 1. Berkowitz RJ, Moss M, Billings RJ, Weinstein P. Clinical outcomes for nursing caries treated using general anesthesia. ASDC J Dent Child 1997; 64(3):210-1, 228. | Title not interest |
| 1. Berkowitz RJ. Causes, treatment and prevention of early childhood caries: a microbiologic perspective. J Can Dent Assoc 2003; 69(5):304-307. | Title not interest |
| 1. Berman MH. Baby bottle caries. Diagnosis and treatment. CDS Rev 1973; 66(2):32-33. | Letter |
| 1. Berman MH. The open-faced crown revisited. CDS Rev 1983; 76(7):21-23. | Case report |
| 1. Bernard-Bonnin AC, Pelletier H, Turgeon JP, Allard-Dansereau C, Petit N, Chabot G et al. Cariogenic feeding habits and fluoride supplementation during infancy and early childhood. Can J Public Health 1993; 84(2):90-93. | Different outcome |
| 1. Bernick SM. What the pediatrician should know about children's teeth. IV. Baby-bottle syndrome. Clin Pediatr (Phila) 1971; 10(4):243-244. | Patient’s hand out |
| 1. Bian JY, Li RY, Wang WJ. Feasibility of milk fluoridation and trends in dental caries of children in China. Adv Dent Res 1995; 9(2):112-115. | Different etiology |
| 1. Bian Z, Du M, Bedi R, Holt R, Jin H, Fan M. Caries experience and oral health behavior in Chinese children with cleft lip and/or palate. Pediatr Dent 2001; 23(5):431-434. | Different etiology |
| 1. Bier-Katz G. ["Baby-bottle syndrome". Overview and therapy]. Zahnarztl Mitt 1982; 72(17):1843-1845. | Language other than English other than English |
| 1. Blake F, Blessmann M, Werle H, Li L, Gbara A. Long-chain 3-hydroxyacyl-CoA dehydrogenase deficiency with inadvertent caries in infants. Int J Paediatr Dent 2007; 17(1):72-74. | Case reports |
| 1. Blanck HM, Marcus M, Tolbert PE, Rubin C, Henderson AK, Hertzberg VS et al. Age at menarche and tanner stage in girls exposed in utero and postnatally to polybrominated biphenyl. Epidemiology 2000; 11(6):641-647. | Title not interest |
| 1. Blanck HM, Marcus M, Tolbert PE, Rubin C, Henderson AK, Hertzberg VS et al. Age at menarche and tanner stage in girls exposed in utero and postnatally to polybrominated biphenyl. Epidemiology 2000; 11(6):641-647. | Title not interest |
| 1. Blanck HM, Marcus M, Hertzberg V, Tolbert PE, Rubin C, Henderson AK et al. Determinants of polybrominated biphenyl serum decay among women in the Michigan PBB cohort. Environ Health Perspect 2000; 108(2):147-152. | Title not interest |
| 1. Blumberg ML, Kunken FR. The dentist's involvement with child abuse. N Y State Dent J 1981; 47(2):65-69. | Case reports |
| 1. Bodenmann P, Madrid C, Vannotti M, Rossi I, Ruiz J. [Migration without borders, but...barriers of meaning]. Rev Med Suisse 2007; 3(135):2710-2717. | Language other than English other than English |
| 1. Bowen WH, Pearson SK, Rosalen PL, Miguel JC, Shih AY. Assessing the cariogenic potential of some infant formulas, milk and sugar solutions. J Am Dent Assoc 1997; 128(7):865-871. | In vitro study |
| 1. Bowen WH. Response to Seow: biological mechanisms of early childhood caries. Community Dent Oral Epidemiol 1998; 26(1 Suppl):28-31. | In vitro study |
| 1. Brice DM, Blum JR, Steinberg BJ. The etiology, treatment, and prevention of nursing caries. Compend Contin Educ Dent 1996; 17(1):92, 94, 96-92, 94, 98. | Review |
| 1. Brothwell DJ, Limeback H. Fluorosis risk in grade 2 students residing in a rural area with widely varying natural fluoride. Community Dentistry and Oral Epidemiology 1999; 27(2):130-136. | Title not interest |
| 1. Brown JP, Junner C, Liew V. A study of Streptococcus mutans levels in both infants with bottle caries and their mothers. Aust Dent J 1985; 30(2):96-98. | Different etiology |
| 1. Bruerd B, Kinney MB, Bothwell E. Preventing baby bottle tooth decay in American Indian and Alaska native communities: a model for planning. Public Health Rep 1989; 104(6):631-640. | Health promotion |
| 1. Bruerd B, Jones C. Preventing baby bottle tooth decay: eight-year results. Public Health Rep 1996; 111(1):63-65. | Health promotion |
| 1. Brugman E, Verrips GH, Danz MJ, Kalsbeek H. Dental prevention among Turkish and Moroccan parents in The Hague. Community Dent Health 1998; 15(2):109-114. | Health promotion |
| 1. Bryant S, McLaughlin K, Morgaine K, Drummond B. Elite athletes and oral health. Int J Sports Med 2011; 32(9):720-724. | Title not interest |
| 1. Buhl S, Wetzel WE, Bodeker RH. [Studies on the incidence of caries in 6- to 48-month old infants]. Dtsch Zahnarztl Z 1989; 44(9):673-677. | Different outcome |
| 1. Cadena GA, Llarena ME, Perez LS, Ojeda LS. [Nursing bottle caries]. Pract Odontol 1987; 8(1):6-2. | Language other than English other than English |
| 1. Cahuana CA, Capella CJ, Cerda E, I. [Polycaries in temporal dentition: a continuing problem]. An Esp Pediatr 1997; 46(3):229-232. | Different etiology |
| 1. Callanan DL, Hiner LB. Vulnerable sibling: hyponatremia from caries prevention. Pediatrics 1987; 79(4):637-639. | Title not interest |
| 1. Campus G, Solinas G, Strohmenger L, Cagetti MG, Senna A, Minelli L et al. National pathfinder survey on children's oral health in Italy: pattern and severity of caries disease in 4-year-olds. Caries Res 2009; 43(2):155-162. | Different etiology |
| 1. Canfield LM, Giuliano AR, Neilson EM, Blashil BM, Graver EJ, Yap HH. Kinetics of the response of milk and serum beta-carotene to daily beta-carotene supplementation in healthy, lactating women. Am J Clin Nutr 1998; 67(2):276-283. | Title not interest |
| 1. Cardash Z, Lahav O, Sarnat H. Complete dentures for the preschool child. Refuat Hashinayim 1991; 9(3):3-10. | Case reports |
| 1. Cartwright A. Breast is best. Br Dent J 2008; 204(7):351-352. | Letter |
| 1. Casamassimo PS. Excerpt from Contemporary Nutrition. ASDC J Dent Child 1978; 45(6):492. | Letter |
| 1. Casamassimo PS. Maternal oral health. Dent Clin North Am 2001; 45(3):469-4vi. | Review |
| 1. Castano FA. Night-bottle syndrome. Pa Dent J (Harrisb ) 1972; 39(1):8-11. | Letter |
| 1. Castilho SD, Rocha MA. Pacifier habit: history and multidisciplinary view. J Pediatr (Rio J ) 2009; 85(6):480-489. | Review |
| 1. Caufield PW, Li Y, Bromage TG. Hypoplasia-associated severe early childhood caries--a proposed definition. J Dent Res 2012; 91(6):544-550. | Review |
| 1. Chaffee BW, Feldens CA, Vitolo MR. Cluster-randomized trial of infant nutrition training for caries prevention. J Dent Res 2013; 92(7 Suppl):29S-36S. | Health promotion |
| 1. Chan-Yip A. Health promotion and research in the Chinese community in Montreal: A model of culturally appropriate health care. Paediatr Child Health 2004; 9(9):627-629. | Title not interest |
| 1. Chedid RN, Bourgeois D, Kaloustian H, Baba N, Pilipili C. Caries prevalence and caries risk in a sample of Lebanese preschool children. Odontostomatol Trop 2011; 34(135):31-45. | Different etiology |
| 1. Chestnutt IG, Murdoch C, Robson KF. Parents and carers' choice of drinks for infants and toddlers, in areas of social and economic disadvantage. Community Dent Health 2003; 20(3):139-145. | Health promotion |
| 1. Ching B, Fujioka C. Comprehensive approach to the management and prevention of early childhood caries. Hawaii Dent J 2003; 34(3):11-12. | Review |
| 1. Chisick MC, Richter P, Piotrowski MJ. Put more "bite" into health promotion: a campaign to revitalize health promotion in the Army Dental Care System. Part I. The mouthguard, sealant, and nursing caries initiatives. Mil Med 2000; 165(8):598-603. | Review |
| 1. Christensen JC. Multiple sclerosis: some epidemiological clues to etiology. Acta Neurol Latinoam 1975; 21(1-4):66-85. | Title not interest |
| 1. Chussid S. Optimizing infant and toddler oral health. The importance of early intervention. Dent Today 2003; 22(7):122-125. | Review |
| 1. Cinar DN. The advantages and disadvantages of pacifier use. Contemp Nurse 2004; 17(1-2):109-112. | Review |
| 1. Cobo E. Characteristics of the Spontaneous Milk Ejecting Activity Occurring During Human Lactation. Journal of Perinatal Medicine 1993; 21(1):77-85. | Title not interest |
| 1. Cockle KL, Bodrati A. Nesting of the White-Throated Woodcreeper Xiphocolaptes Albicollis. Wilson Journal of Ornithology 2013; 125(4):782-789. | Title not interest |
| 1. Colak H, Dulgergil CT, Dalli M, Hamidi MM. Early childhood caries update: A review of causes, diagnoses, and treatments. J Nat Sci Biol Med 2013; 4(1):29-38 | Review |
| 1. Cone TE, Jr. The nursing bottle caries syndrome. JAMA 1981; 245(22):2334. | Case reports |
| 1. Contreras B, Ewan C. A culturally-specific oral health program for high risk Vietnamese children. Probe 1996; 30(4):156-157. | Title not interest |
| 1. Cook HW, Duncan WK, De BS, Berg B. The cost of nursing caries in a Native American Head Start population. J Clin Pediatr Dent 1994; 18(2):139-142. | Different outcome |
| 1. Cooke ME, Davidson LE, Livesey SL. Schinzel-Giedion syndrome: interesting facial and orodental features, and dental management. Int J Paediatr Dent 2002; 12(1):66-72. | Case reports |
| 1. Corcellas C, Feo ML, Torres JP, Malm O, Ocampo-Duque W, Eljarrat E et al. Pyrethroids in human breast milk: occurrence and nursing daily intake estimation. Environ Int 2012; 47:17-22. | Title not interest |
| 1. Correa-Faria P, Martins PA, Vieira-Andrade RG, Marques LS, Ramos-Jorge ML. Perinatal factors associated with developmental defects of enamel in primary teeth: a case-control study. Brazilian Oral Research 2013; 27(4):363-368. | Title not interest |
| 1. Correa-Faria P, Martins PA, Vieira-Andrade RG, Oliveira-Ferreira F, Marques LS, Ramos-Jorge ML. Developmental defects of enamel in primary teeth: prevalence and associated factors. International Journal of Paediatric Dentistry 2013; 23(3):173-179. | Title not interest |
| 1. Crawford JG, Testa RG, Stone BC. Breast feeding vs. bottle feeding as related to dental caries incidence: a review of the literature. Refuat Hapeh Vehashinayim 1974; 23(0):19-26. | Review |
| 1. Croll TP. The need for early infant and toddler dental care: a pictorial argument. Quintessence Int 1988; 19(10):719-730. | Letter |
| 1. Crow DR. Baby bottle tooth decay prevention--a new program for the Texas Department of Health. Tex Dent J 1992; 109(8):141. | Health promotion |
| 1. Currier GF, Glinka MP. The prevalence of nursing bottle caries or baby bottle syndrome in an inner city fluoridated community. Va Dent J 1977; 54(5):9-19. | Review |
| 1. Curzon ME. "Out of the mouths of babes and sucklings...." ...do we still care for the oral health of our children? J Dent Res 1994; 73(4):714-715. | Editoral |
| 1. Curzon ME, Preston AJ. Risk groups: nursing bottle caries/caries in the elderly. Caries Res 2004; 38 Suppl 1:24-33. | Review |
| 1. Dalben GD, Costa B, Gomide MR, Das Neves LT. Breast-feeding and sugar intake in babies with cleft lip and palate. Cleft Palate-Craniofacial Journal 2003; 40(1):84-87. | Different etiology |
| 1. Daly B, Sharif MO, Newton T, Jones K, Worthington H, V. Local interventions for the management of alveolar osteitis (dry socket). Cochrane Database of Systematic Reviews 2012. | Title not interest |
| 1. Dasanayake AP, Caufield PW. Prevalence of dental caries in Sri Lankan aboriginal Veddha children. Int Dent J 2002; 52(6):438-444. | In vitro study |
| 1. Davies AN, Shorthose K. Parasympathomimetic drugs for the treatment of salivary gland dysfunction due to radiotherapy. Cochrane Database of Systematic Reviews 2007. | Title not interest |
| 1. Davies GM, Duxbury JT, Boothman NJ, Davies RM, Blinkhorn AS. A staged intervention dental health promotion programme to reduce early childhood caries. Community Dent Health 2005; 22(2):118-122. | Health Promotion |
| 1. Davies GN. Early childhood caries--a synopsis. Community Dent Oral Epidemiol 1998; 26(1 Suppl):106-116. | Review |
| 1. Dawidowicz A, Krajewska K, Krajewska-Kulak E, Kulikowski M, Szyszko-Perlowska A, Rolka H et al. [Women's knowledge of health behaviors in the puerperium]. Wiad Lek 2004; 57 Suppl 1:70-73. | Title not interest |
| 1. de Soet JJ, Kreulen CM, Veerkamp JS, Bokhout B, van LC, de GJ. Transmission of "Streptococcus mutans" in nursing bottle caries and cleft palate patients. Adv Exp Med Biol 1997; 418:181-183. | Title not interest |
| 1. DenBesten P, Berkowitz R. Early childhood caries: an overview with reference to our experience in California. J Calif Dent Assoc 2003; 31(2):139-143. | Review |
| 1. Dennison BA. Fruit juice consumption by infants and children: a review. J Am Coll Nutr 1996; 15(5 Suppl):4S-11S. | Review |
| 1. Dilley GJ, Dilley DH, Machen JB. Prolonged nursing habit: a profile of patients and their families. ASDC J Dent Child 1980; 47(2):102-108. | Different outcome |
| 1. Dorschug H. [Tongue, teeth, sweet tea. Orthodontic preventative car]. Kinderkrankenschwester 2004; 23(5):195-199. | Title not interest |
| 1. Douglass JM, O'Sullivan DM, Tinanoff N. Temporal changes in dental caries levels and patterns in a Native American preschool population. J Public Health Dent 1996; 56(4):171-175. | Title not interest |
| 1. Drinkard C, Dilley DC. Rampant caries as a result of a bizarre food habit: a case report. Pediatr Dent 1982; 4(2):131-134. | Case report |
| 1. Dube VK, Berg J. "Pediatric dentistry today". J Gt Houst Dent Soc 1995; 66(7):27-34. | Editoral |
| 1. Duggal MS, van LC. Dental considerations for dietary counselling. Int Dent J 2001; 51(6 Suppl 1):408-412. | Review |
| 1. Dunson DB, Chulada P, Arbes SJ, Jr. Bayesian modeling of time-varying and waning exposure effects. Biometrics 2003; 59(1):83-91. | Title not interest |
| 1. Eigbobo JO, Onyeaso CO. Maternal knowledge and awareness of factors affecting oral health in the paediatric population. Odontostomatol Trop 2013; 36(142):15-24. | Title not interest |
| 1. Ekstrand J, Hardell LI, Spak CJ. Fluoride balance studies on infants in a 1-ppm-water-fluoride area. Caries Res 1984; 18(1):87-92. | Title not interest |
| 1. Emanuelsson IM. Mutans streptococci--in families and on tooth sites. Studies on the distribution, acquisition and persistence using DNA fingerprinting. Swed Dent J Suppl 2001;(148):1-66. | Title not interest |
| 1. Emanuelsson IMR, Wang XM. Demonstration of identical strains of mutans streptococci within Chinese families by genotyping. European Journal of Oral Sciences 1998; 106(3):788-794. | Title not interest |
| 1. Emanuelsson IR, Li Y, Bratthall D. Genotyping shows different strains of mutans streptococci between father and child and within parental pairs in Swedish families. Oral Microbiol Immunol 1998; 13(5):271-277. | Title not interest |
| 1. Ericsson Y, Ribelius U. Wide variations of fluoride supply to infants and their effect. Caries Res 1971; 5(1):78-88. | Title not interest |
| 1. Ericsson Y. Effect of infant diets with widely different fluoride contents on the fluoride concentrations of deciduous teeth. Caries Res 1973; 7(1):56-62. | Title not interest |
| 1. Evans S. Development of an infant feeding policy in southern Derbyshire. Health Visit 1995; 68(2):59-60. | Different outcome |
| 1. Everhart DL, Klapper B, Carter WH, Jr., Moss S. Evaluation of dental caries experiences and salivary IgA in children ages 3-7. Caries Res 1977; 11(4):211-215. | Title not interest |
| 1. Fadavi S. Management of early childhood caries. Gen Dent 2003; 51(1):38-40. | Editorial |
| 1. Faine MP, Oberg D. Snacking and oral health habits of Washington state WIC children and their caregivers. ASDC J Dent Child 1994; 61(5-6):350-355. | Different outcome |
| 1. Faine MP, Oberg D. Survey of dental nutrition knowledge of WIC nutritionists and public health dental hygienists. J Am Diet Assoc 1995; 95(2):190-194. | Title not interest |
| 1. Feigal RJ. Common oral diseases of children. Pediatr Ann 1985; 14(2):133-138. | Title not interest |
| 1. Feldens CA, Vitolo MR, Drachler ML. A randomized trial of the effectiveness of home visits in preventing early childhood caries. Community Dent Oral Epidemiol 2007; 35(3):215-223. | Health promotion |
| 1. Feldens CA, Kramer PF, Sequeira MC, Rodrigues PH, Vitolo MR. Maternal education is an independent determinant of cariogenic feeding practices in the first year of life. Eur Arch Paediatr Dent 2012; 13(2):70-75. | Different etiology |
| 1. Fieguth A, Gunther D, Kleemann WJ, Troger HD. Lethal child neglect. Forensic Sci Int 2002; 130(1):8-12. | Title not interest |
| 1. Finlayson TL, Siefert K, Ismail AI, Delva J, Sohn W. Reliability and validity of brief measures of oral health-related knowledge, fatalism, and self-efficacy in mothers of African American children. Pediatr Dent 2005; 27(5):422-428. | Title not interest |
| 1. Flynn FW, Culver B, Newton SV. Salt intake by normotensive and spontaneously hypertensive rats: two-bottle and lick rate analyses. Physiol Behav 2003; 78(4-5):689-696. | Title not interest |
| 1. Fomon SJ, Ekstrand J. Fluoride intake by infants. Journal of Public Health Dentistry 1999; 59(4):229-234. | Title not interest |
| 1. Fomon SJ, Ekstrand J, Ziegler EE. Fluoride intake and prevalence of dental fluorosis: Trends in fluoride intake with special attention to infants. Journal of Public Health Dentistry 2000; 60(3):131-139. | Title not interest |
| 1. Fontana M, Catt D, Eckert GJ, Ofner S, Toro M, Gregory RL et al. Xylitol: effects on the acquisition of cariogenic species in infants. Pediatr Dent 2009; 31(3):257-266. | Title not interest |
| 1. Foreman FJ, Theobald WD. Direct bonded glass ionomer crowns. ASDC J Dent Child 1987; 54(3):165-169. | Case reports |
| 1. Forsman B. Dental fluorosis and caries in high-fluoride districts in Sweden. Community Dent Oral Epidemiol 1974; 2(3):132-148. | Title not interest |
| 1. Fracasso ML, Rios D, Provenzano MG, Goya S. Efficacy of an oral health promotion program for infants in the public sector. J Appl Oral Sci 2005; 13(4):372-376. | Health promotion |
| 1. Franco S, Theriot J, Greenwell A. The influence of early counselling on weaning from a bottle. Community Dent Health 2008; 25(2):115-118. | Health promotion |
| 1. Freeman R, Stevens A. Nursing caries and buying time: an emerging theory of prolonged bottle feeding. Community Dent Oral Epidemiol 2008; 36(5):425-433. | Different outcome |
| 1. Frigoletto R. Simplified treatment of bottle baby syndrome. ASDC J Dent Child 1976; 43(2):90-91. | Editorial |
| 1. Frigoletto RL. Update--simplified treatment of bottle-baby syndrome. ASDC J Dent Child 1982; 49(5):374-376. | Editorial |
| 1. Fuhlrott E. [Diabolical circle of caries prophylaxis]. Zahnarztl Mitt 1971; 61(21):1064-1067. | Title not interest |
| 1. Furness S, Worthington H, V, Bryan G, Birchenough S, McMillan R. Interventions for the management of dry mouth: topical therapies. Cochrane Database of Systematic Reviews 2011. | Title not interest |
| 1. Galijasevic S, Maitra D, Lu T, Sliskovic I, Abdulhamid I, Abu-Soud HM. Myeloperoxidase interaction with peroxynitrite: chloride deficiency and heme depletion. Free Radical Biology and Medicine 2009; 47(4):431-439. | Title not interest |
| 1. Gandini P, Schiavi A, Camassa D, Manuelli M. [Statistical survey of malocclusion in school age children]. Mondo Ortod 1989; 14(1):73-78. | Title not interest |
| 1. Gardner DE, Norwood JR, Eisenson JE. At-will breast feeding and dental caries: four case reports. ASDC J Dent Child 1977; 44(3):186-191. | Case reports |
| 1. Gehrke FS, Johnsen DS. Bottle caries associated with anti-HIV therapy. Pediatr Dent 1991; 13(1):73. | Case reports/Letter |
| 1. Gizani S, Vinckier F, Declerck D. Caries pattern and oral health habits in 2- to 6-year-old children exhibiting differing levels of caries. Clin Oral Investig 1999; 3(1):35-40. | Different etiology |
| 1. Gooze RA, Anderson SE, Whitaker RC. Prolonged Bottle Use and Obesity at 5.5 Years of Age in US Children. Journal of Pediatrics 2011; 159(3):431-436. | Different outcome |
| 1. Gratrix D, Holloway PJ. Factors of deprivation associated with dental caries in young children. Community Dent Health 1994; 11(2):66-70. | Different ethiology |
| 1. Griffen AL, Goepferd SJ. Preventive oral health care for the infant, child, and adolescent. Pediatr Clin North Am 1991; 38(5):1209-1226. | Review |
| 1. Grindefjord M, Dahllof G, Wikner S, Hojer B, Modeer T. Prevalence of mutans streptococci in one-year-old children. Oral Microbiol Immunol 1991; 6(5):280-283. | Different etiology |
| 1. Gudino S, Rojas N, Castro C, Rodriguez M, Vega M, Lopez LM. Colonization of mutans streptococci in Costa Rican children from a high-risk population. J Dent Child (Chic ) 2007; 74(1):36-40. | Different etiology |
| 1. Gulec SA, Siegel JA. Posttherapy radiation safety considerations in radiomicrosphere treatment with 90Y-microspheres. J Nucl Med 2007; 48(12):2080-2086 | Title not interest |
| 1. Hallett KB, O'Rourke PK. Social and behavioural determinants of early childhood caries. Aust Dent J 2003; 48(1):27-33. | Different outcome |
| 1. Hallett KB, O'Rourke PK. Caries experience in preschool children referred for specialist dental care in hospital. Aust Dent J 2006; 51(2):124-129. | Different etiology |
| 1. Hamilton FA, Davis KE, Blinkhorn AS. An oral health promotion programme for nursing caries. Int J Paediatr Dent 1999; 9(3):195-200. | Health promotion |
| 1. Hansen MK. From the president. ASDC J Dent Child 1978; 45(1):4. | Letter |
| 1. Hardison JD, Cecil JC, White JA, Manz M, Mullins MR, Ferretti GA. The 2001 Kentucky Childrens Oral Health Survey: findings for children ages 24 to 59 months and their caregivers. Pediatr Dent 2003; 25(4):365-372. | Different etiology |
| 1. Harrison RL, Wong T. An oral health promotion program for an urban minority population of preschool children. Community Dent Oral Epidemiol 2003; 31(5):392-399. | Different etiology |
| 1. Hart HM. Use of dummies, reservoir feeders, and comforters in a child population in North London. Lancet 1969; 2(7611):99-101. | Title not interest |
| 1. Hashida S, Mihara J, Hashida K, Sumi N, Rakugi M, Ooshima T et al. [Clinical and statistical survey of the children at the Pedodontic Clinic of the Osaka University Dental Hospital]. Osaka Daigaku Shigaku Zasshi 1985; 30(2):336-344. | Title not interest |
| 1. Hashim Nainar SM. Nursing cFaries: an overview. J Conn State Dent Assoc 1990; 66(2):34-37. | Review |
| 1. Haskins DR. Pediatric dental rehabilitation procedures in the OR. AORN J 1996; 64(4):573-579. | Review |
| 1. Hata H, Horiuchi M, Toba M, Hata S, Tsukada M, Mayanagi H. Lactose-fermenting bacteria from caries lesions of breast fed children. Journal of Dental Research 1998; 77:138. | Different etiology |
| 1. Hattab FN, Al-Omari MA, Angmar-Mansson B, Daoud N. The prevalence of nursing caries in one-to-four-year-old children in Jordan. ASDC J Dent Child 1999; 66(1):53-58. | Different etiology |
| 1. Hein W. [Correct dosage of fluorides will strengthen the tooth germs]. Zahnarztl Mitt 1979; 69(19):1180-1182. | Title not interest |
| 1. Heine W, Braun OH, Mohr C, Leitzmann P. Enhancement of lysozyme trypsin-mediated decay of intestinal bifidobacteria and lactobacilli. J Pediatr Gastroenterol Nutr 1995; 21(1):54-58. | Title not interest |
| 1. Helderman WHV, Mabelya L, vantHof MA, Konig KG. Two types of intraoral distribution of fluorotic enamel. Community Dentistry and Oral Epidemiology 1997; 25(3):251-255. | Title not interest |
| 1. Henderson HZ, Dean JA, Hatcher EA. Indiana infant-toddler dental care survey. J Indiana Dent Assoc 1991; 70(1):8-13. | Different outcome |
| 1. Hicks RJ, Binns D, Stabin MG. Pattern of uptake and excretion of (18)F-FDG in the lactating breast. J Nucl Med 2001; 42(8):1238-1242. | Title not interest |
| 1. Hicks TW, Davis-Burchat L, Fendley S, Jeffrey B. Infant feeding caries: Part II. The Simcoe and Muskoka-Parry Sound Health Unit Project. Ont Dent 1995; 72(9):24-6, 33. | Title not interest |
| 1. Hicks TW, Davis-Burchat L, Fendley S, Jeffrey B. Infant feeding caries: Part I. A review and trial preventive project. Ont Dent 1995; 72(9):17-3. | Review |
| 1. Himelhoch DA, Mostofi R. Oral abnormalities in the Ellis-van Creveld syndrome: case report. Pediatr Dent 1988; 10(4):309-313. | Case Report |
| 1. Holgerson PL, Vestman NR, Claesson R, Ohman C, Domellof M, Tanner AC et al. Oral microbial profile discriminates breast-fed from formula-fed infants. J Pediatr Gastroenterol Nutr 2013; 56(2):127-136. | Different etiology |
| 1. Holloway GL, Caspersen JP, Vanderwel MC, Naylor BJ. Cavity tree occurrence in hardwood forests of central Ontario. Forest Ecology and Management 2007; 239(1-3):191-199. | Title not interest |
| 1. Holm AK, Andersson R. Enamel mineralization disturbances in 12-year-old children with known early exposure to fluorides. Community Dent Oral Epidemiol 1982; 10(6):335-339. | Title not interest |
| 1. Holt RD, Joels D, Winter GB. Caries in pre-school children. The Camden study. Br Dent J 1982; 153(3):107-109. | Title not interest |
| 1. Horton S, Barker JC. Rural Latino immigrant caregivers' conceptions of their children's oral disease. J Public Health Dent 2008; 68(1):22-29. | Title not interest |
| 1. Hossny E, Reda S, Marzouk S, Diab D, Fahmy H. Serum fluoride levels in a group of Egyptian infants and children from Cairo city. Arch Environ Health 2003; 58(5):306-315. | Title not interest |
| 1. Hunter ML, Hood CA, Hunter B, Kingdon A. Reported infant feeding, oral hygiene and dental attendance patterns in children aged 5 years and under referred for extraction of teeth under general anaesthesia. Int J Paediatr Dent 1997; 7(4):243-248. | Title not interest |
| 1. Imbeau L, Desrochers A. Foraging ecology and use of drumming trees by three-toed woodpeckers. Journal of Wildlife Management 2002; 66(1):222-231. | Title not interest |
| 1. Inoue N. [Lactation and ablactation periods]. Shikai Tenbo 1985; 65(5):1093-1098. | Title not interest |
| 1. Jaafar SH, Jahanfar S, Angolkar M, Ho JJ. Effect of restricted pacifier use in breastfeeding term infants for increasing duration of breastfeeding. Cochrane Database of Systematic Reviews 2012. | Different outcome |
| 1. Jasmin JR. [Nursing bottle caries]. Pedod Fr 1978; 12:203-209. | Language other than English other than English |
| 1. John J. Home visits for dietary advice reduce caries. Evid Based Dent 2008; 9(1):11. | Editorial |
| 1. Johnsen DC. The role of the pediatrician in identifying and treating dental caries. Pediatr Clin North Am 1991; 38(5):1173-1181. | Comment |
| 1. Johnsen DC. Response to Horowitz: research issues in early childhood caries. Community Dent Oral Epidemiol 1998; 26(1 Suppl):82-83. | Comment |
| 1. Johnson KR. Restorations for bottle-mouth syndrome and fractured anterior teeth. Dent Surv 1980; 56(1):30-33. | Case reports |
| 1. Johnston T, Messer LB. Nursing caries: literature review and report of a case managed under local anaesthesia. Aust Dent J 1994; 39(6):373-381. | Review |
| 1. Juambeltz JC, Kula K, Perman J. Nursing caries and lactose intolerance. ASDC J Dent Child 1993; 60(4):377-384. | Title not interest |
| 1. Kabus K. [Tea for children and the baby bottle]. SSO Schweiz Monatsschr Zahnheilkd 1982; 92(12):1138-1141. | Title not interest |
| 1. Kacho MA, Zahedpasha Y, Eshkevari P. Comparison of the rate of exclusive breast-feeding between pacifier sucker and non-sucker infants. Iranian Journal of Pediatrics 2007; 17(2):113-117. | Different outcome |
| 1. Kahn R, Bonuck K, Trombley M. Randomized controlled trial of bottle weaning intervention: a pilot study. Clin Pediatr (Phila) 2007; 46(2):163-174. | Different outcome |
| 1. Kammerman AM, Starkey PE. Nursing caries: a case history. J Indiana Dent Assoc 1981; 60(4):7-10. | Case report |
| 1. Kanellis MJ, Logan HL, Jakobsen J. Changes in maternal attitudes toward baby bottle tooth decay. Pediatr Dent 1997; 19(1):56-60. | Title not interest |
| 1. Kanou N, Koseki A, Yamada K, Sakurai S, Ohnishi N, Mayanagi H et al. [Investigation into the actual condition of outpatients. II. Correlation between the daily habits of eating and toothbrushing and the prevalence of dental caries incidence]. Shoni Shikagaku Zasshi 1989; 27(2):467-474. | Title not interest |
| 1. Kanwar JR, Kanwar RK, Sun X, Punj V, Matta H, Morley SM et al. Molecular and biotechnological advances in milk proteins in relation to human health. Curr Protein Pept Sci 2009; 10(4):308-338. | Title not interest |
| 1. Karmaus W, Fussman C, Muttineni J, Zhu X. Backward estimation of exposure to organochlorines using repeated measurements. Environ Health Perspect 2004; 112(6):710-716. | Title not interest |
| 1. Karn TA, O'Sullivan DM, Tinanoff N. Colonization of mutans streptococci in 8- to 15-month-old children. J Public Health Dent 1998; 58(3):248-249. | Title not interest |
| 1. Karp WB. Childhood and adolescent obesity: a national epidemic. J Calif Dent Assoc 1998; 26(10):771-773. | Title not interest |
| 1. Kassab M, Foster JP, Foureur M, Fowler C. Sweet-tasting solutions for needle-related procedural pain in infants one month to one year of age. Cochrane Database of Systematic Reviews 2012. | Title not interest |
| 1. Kaste LM, Marianos D, Chang R, Phipps KR. The assessment of nursing caries and its relationship to high caries in the permanent dentition. J Public Health Dent 1992; 52(2):64-68. | Different outcome |
| 1. Kaste LM, Gift HC. Inappropriate infant bottle feeding. Status of the Healthy People 2000 objective. Arch Pediatr Adolesc Med 1995; 149(7):786-791. | Different outcome |
| 1. Kaste LM, Marianos D, Chang R, Phipps KR. The assessment of nursing caries and its relationship to high caries in the permanent dentition. 1992. J Indiana Dent Assoc 2010; 89(2):20-24. | Title not interest |
| 1. Katz L, Ripa LW, Petersen M. Nursing caries in Head Start children, St. Thomas U.S. Virgin Islands: assessed by examiners with different dental backgrounds. J Clin Pediatr Dent 1992; 16(2):124-128. | Different etiology |
| 1. Katzenberg MA, Saunders SR, Fitzgerald WR. Age differences in stable carbon and nitrogen isotope ratios in a population of prehistoric maize horticulturists. Am J Phys Anthropol 1993; 90(3):267-281 | Title not interest |
| 1. Kawabata K, Kawamura M, Sasahara H, Morishita M, Bachchu MA, Iwamoto Y. Development of an oral health indicator in infants. Community Dent Health 1997; 14(2):79-83. | Different etiology |
| 1. Kellerhoff NM, Lussi A. ["Molar-incisor hypomineralization"]. Schweiz Monatsschr Zahnmed 2004; 114(3):243-253. | Title not interest |
| 1. Kelly M, Bruerd B. The prevalence of baby bottle tooth decay among two native American populations. J Public Health Dent 1987; 47(2):94-97. | Different etiology |
| 1. Kelsey RG, Hennon PE, Huso M, Karchesy JJ. Changes in heartwood chemistry of dead yellow-cedar trees that remain standing for 80 years or more in southeast Alaska. J Chem Ecol 2005; 31(11):2653-2670. | Title not interest |
| 1. Kendrick F, Wilson S, Coury DL, Preisch JW. Comparison of temperaments of children with and without baby bottle tooth decay. ASDC J Dent Child 1998; 65(3):198-203. | Title not interest |
| 1. King DL, Leimone CA. Nursing bottle caries--a preventive dilemma. Dent Assist 1978; 47(5):18-19. | Editorial |
| 1. Kobayashi HM, Scavone H, Ferreira RI, Garib DG. Relationship between breastfeeding duration and prevalence of posterior crossbite in the deciduous dentition. American Journal of Orthodontics and Dentofacial Orthopedics 2010; 137(1):54-58. | Different outcome |
| 1. Konig KG. Diet and oral health. International Dental Journal 2000; 50(3):162-174. | Review |
| 1. Koranyi K, Rasnake LK, Tarnowski KJ. Nursing bottle weaning and prevention of dental caries: a survey of pediatricians. Pediatr Dent 1991; 13(1):32-34. | Different etiology |
| 1. Koroluk LD, Riekman GA. Parental perceptions of the effects of maxillary incisor extractions in children with nursing caries. ASDC J Dent Child 1991; 58(3):233-236. | Quality health |
| 1. Koseki A, Kanou N, Yamada K, Sakurai S, Ohnishi N, Mayanagi H et al. [Investigation into the actual condition of outpatients. I. The environmental and oral health status prior to and at the first visit. Comparison of the results between present study and last investigation]. Shoni Shikagaku Zasshi 1989; 27(2):457-466. | Title not interest |
| 1. Kotlow LA. The influence of the maxillary frenum on the development and pattern of dental caries on anterior teeth in breastfeeding infants: prevention, diagnosis, and treatment. J Hum Lact 2010; 26(3):304-308. | Different etiology |
| 1. Kowash MB, Pinfield A, Smith J, Curzon ME. Effectiveness on oral health of a long-term health education programme for mothers with young children. Br Dent J 2000; 188(4):201-205. | Health promotion |
| 1. Kramer MS, Matush L, Bogdanovich N, Aboud F, Mazer B, Fombonne E et al. Health and development outcomes in 6.5-y-old children breastfed exclusively for 3 or 6 mo. Am J Clin Nutr 2009; 90(4):1070-1074. | Different outcome |
| 1. Kreulen CM, de Soet HJ, Hogeveen R, Veerkamp JS. Streptococcus mutans in children using nursing bottles. ASDC J Dent Child 1997; 64(2):107-111. | Different etiology |
| 1. Krishnakumar R, Singh S, Subba Reddy VV. Comparison of levels of mutans streptococci and lactobacilli in children with nursing bottle caries, rampant caries, healthy children with 3-5 dmft/DMFT and healthy caries free children. J Indian Soc Pedod Prev Dent 2002; 20(1):1-5. | Different etiology |
| 1. Kristjansson B, Petticrew M, MacDonald B, Krasevec J, Janzen L, Greenhalgh T et al. School feeding for improving the physical and psychosocial health of disadvantaged students. Cochrane Database of Systematic Reviews 2007. | Title not interest |
| 1. Kristjansson E, Francis DK, Liberato S, Benkhalti JM, Welch V, Batal M et al. Feeding interventions for improving the physical and psychosocial health of disadvantaged children aged three months to five years. Cochrane Database of Systematic Reviews 2012. | Title not interest |
| 1. Ksykiewicz-Dorota A, Kaminska B. Health care reform and the scope of independence in decision making by environmental/family nurses. III. New concept of health care and currently provided scope of services. Ann Univ Mariae Curie Sklodowska Med 2003; 58(2):294-299. | Title not interest |
| 1. Kumari NR, Sheela S, Sarada PN. Knowledge and attitude on infant oral health among graduating medical students in Kerala. J Indian Soc Pedod Prev Dent 2006; 24(4):173-176. | Title not interest |
| 1. Kuokkanen M, Kokkonen J, Enattah NS, Ylisaukko-Oja T, Komu H, Varilo T et al. Mutations in the translated region of the lactase gene (LCT) underlie congenital lactase deficiency. American Journal of Human Genetics 2006; 78(2):339-344. | Title not interest |
| 1. Kuthy RA, Quilty JF, Jr., Levy SM, Benninger G. Dietary fluoride supplements for Ohio children--the role of the physician. Ohio Med 1990; 86(8):613-615. | Title not interest |
| 1. Lane BJ, Sellen V. Bottle caries: a nursing responsibility. Can J Public Health 1986; 77(2):128-130. | Title not interest |
| 1. Law CS, Sheehan M, Needleman HL. Centronuclear myopathy and nursing pattern caries: management of a 1 year old. J Clin Pediatr Dent 1995; 20(1):69-72. | Case report |
| 1. Law CS. The impact of changing parenting styles on the advancement of pediatric oral health. J Calif Dent Assoc 2007; 35(3):192-197. | Different etiology |
| 1. Lee C, Rezaiamira N, Jeffcott E, Oberg D, Domoto P, Weinstein P. Teaching parents at WIC clinics to examine their high caries-risk babies. ASDC J Dent Child 1994; 61(5-6):347-349. | Title not interest |
| 1. Lee CM, Blain SM, Duperon DF. Parents' self-reported compliance with preventive practices after witnessing their child undergo intravenous sedation for dental treatment. ASDC J Dent Child 2002; 69(1):77-80, 13. | Title not interest |
| 1. Lehman DA, Chung MH, Mabuka JM, John-Stewart GC, Kiarie J, Kinuthia J et al. Lower risk of resistance after short-course HAART compared with zidovudine/single-dose nevirapine used for prevention of HIV-1 mother-to-child transmission. J Acquir Immune Defic Syndr 2009; 51(5):522-529. | Title not interest |
| 1. Leppaniemi A, Lukinmaa PL, Alaluusua S. Nonfluoride hypomineralizations in the permanent first molars and their impact on the treatment need. Caries Res 2001; 35(1):36-40. | Title not interest |
| 1. Levoy RP. Let's educate MDs about 'nursing bottle syndrome'. Dent Econ 1976; 66(9):95-98. | Editorial |
| 1. Levy SM, Zarei M. Evaluation of fluoride exposures in children. ASDC J Dent Child 1991; 58(6):467-473. | Title not interest |
| 1. Lewin S, Munabi BS, Glenton C, Daniels K, Bosch C, X, van-Wyk BE et al. Lay health workers in primary and community health care for maternal and child health and the management of infectious diseases. Cochrane Database of Systematic Reviews 2010. | Title not interest |
| 1. Lewis DW, Limeback H. Comparison of recommended and actual mean intakes of fluoride by Canadians. J Can Dent Assoc 1996; 62(9):708-5. | Title not interest |
| 1. Li S, Liu T, Zhuang H. [Detection of the transmitted strains and non-transmitted strains of Mutans streptococci by AP-PCR]. Hua Xi Kou Qiang Yi Xue Za Zhi 2003; 21(5):392-395. | Title not interest |
| 1. Li Y, Navia JM, Caufield PW. Colonization by mutans streptococci in the mouths of 3- and 4-year-old Chinese children with or without enamel hypoplasia. Arch Oral Biol 1994; 39(12):1057-1062. | Different etiology |
| 1. Li Y, Wang W, Caufield PW. The fidelity of mutans streptococci transmission and caries status correlate with breast-feeding experience among Chinese families. Caries Res 2000; 34(2):123-132. | Title not interest |
| 1. Li YH, Navia JM, Caufield PW. Colonization by Mutans-Streptococci in the Mouths of 3-Year-Old and 4-Year-Old Chinese Children with Or Without Enamel Hypoplasia. Archives of Oral Biology 1994; 39(12):1057-1062. | Different etiology |
| 1. Lin YT, Tsai CL. Caries prevalence and bottle-feeding practices in 2-year-old children with cleft lip, cleft palate, or both in Taiwan. Cleft Palate Craniofac J 1999; 36(6):522-526. | Different etiology |
| 1. Litsas G. Effect of full mouth rehabilitation on the amount of Streptococcus mutans in children with Early Childhood Caries. Eur J Paediatr Dent 2010; 11(1):35-38. | Title not interest |
| 1. Liu J. Neglected problem: nursing bottle syndrome. Dentistry (Loma Linda ) 1990; 3(2):57-58. | Case report |
| 1. Liu J, Killilea DW, Ames BN. Age-associated mitochondrial oxidative decay: improvement of carnitine acetyltransferase substrate-binding affinity and activity in brain by feeding old rats acetyl-L- carnitine and/or R-alpha -lipoic acid. Proc Natl Acad Sci U S A 2002; 99(4):1876-1881. | Title not interest |
| 1. Lodi G, Figini L, Sardella A, Carrassi A, Del FM, Furness S. Antibiotics to prevent complications following tooth extractions. Cochrane Database of Systematic Reviews 2012. | Title not interest |
| 1. Loevy HT, Kowitz A. Dentistry on stamps. J Am Dent Assoc 1989; 118(5):609 | Title not interest |
| 1. Logan HL, Baron RS, Kanellis M, Brennan M, Brunsman BA. Knowledge of male and female midwestern college students about baby bottle tooth decay. Pediatr Dent 1996; 18(3):219-223. | Title not interest |
| 1. Lopez del Valle LM, Riedy CA, Weinstein P. Rural Puerto Rican women's views on children's oral health: a qualitative community-based study. J Dent Child (Chic ) 2005; 72(2):61-66. | Title not interest |
| 1. Lopez L, Berkowitz RJ, Moss ME, Weinstein P. Mutans streptococci prevalence in Puerto Rican babies with cariogenic feeding behaviors. Pediatr Dent 2000; 22(4):299-301. | Different etiology |
| 1. Losso EM, Tavares MCR, da Silva JYB, Urban CD. Breastfeeding and early childhood caries: a myth that survives Reply. Jornal de Pediatria 2009; 85(5):465-466. | Letter |
| 1. Love SB. Nursing bottle syndrome. Md State Med J 1979; 28(3):44-45. | Letter |
| 1. Lucan RK, Andreas M, Benda P, Bartonicka T, Brezinova T, Hoffmannova A et al. Alcathoe bat (Myotis alcathoe) in the Czech Republic: distributional status, roosting and feeding ecology. Acta Chiropterologica 2009; 11(1):61-69. | Title not interest |
| 1. Lukes SM. Oral health knowledge attitudes and behaviors of migrant preschooler parents. J Dent Hyg 2010; 84(2):87-93. | Title not interest |
| 1. Lyons SF, Bowers ET, McGillivray GM, Blackburn NK, Gray GE. Evaluation of the MUREX*ICE HIV-1.0.2 capture enzyme immunoassay for early identification of HIV-1 seroreverting infants in a developing country. Clinical and Diagnostic Virology 1997; 8(1):1-8. | Title not interest |
| 1. MacKeown JM, Faber M. Urbanisation and cariogenic food habits among 4-24-month-old black South African children in rural and urban areas. Public Health Nutr 2002; 5(6):719-726. | Different etiology |
| 1. Mackie IC, Blinkhorn AS. Rampant caries revisited. Dent Update 1990; 17(9):390-391. | Case report |
| 1. Magraith KS. Oral health for the preschool child. Med J Aust 1999; 170(9):455-456. | Letter |
| 1. Maltz J. Restoring primary teeth: introducing a new material. Ont Dent 1992; 69(8):36. | Case report |
| 1. Mani SA, Aziz AA, John J, Ismail NM. Knowledge, attitude and practice of oral health promoting factors among caretakers of children attending day-care centers in Kubang Kerian, Malaysia: a preliminary study. J Indian Soc Pedod Prev Dent 2010; 28(2):78-83. | Title not interest |
| 1. Mani SA, Burhanudin NA, John J. Malaysian undergraduates' knowledge and opinions on Early Childhood oral health. Eur J Paediatr Dent 2012; 13(1):64-68. | Title not interest |
| 1. Marchant S, Brailsford SR, Twomey AC, Roberts GJ, Beighton D. The predominant microflora of nursing caries lesions. Caries Res 2001; 35(6):397-406. | Different etiology |
| 1. Martin ND. Optimal fluoride intake. Med J Aust 1973; 1(22):1118 | Title not interest |
| 1. Martinez SL, Diaz GE, Garcia-Tornel FS, Gaspa MJ. [Pacifier use: risks and benefits]. An Esp Pediatr 2000; 53(6):580-585. | Review |
| 1. Masumo R, Bardsen A, Astrom AN. Developmental defects of enamel in primary teeth and association with early life course events: a study of 6-36 month old children in Manyara, Tanzania. Bmc Oral Health 2013; 13. | Different etiology |
| 1. Masumo R, Bardsen A, Astrom AN. Developmental defects of enamel in primary teeth and association with early life course events: a study of 6-36 month old children in Manyara, Tanzania. Bmc Oral Health 2013; 13:21. | Different etiology |
| 1. Matee MI, Mikx FH, Maselle SY, van Palenstein Helderman WH. Rampant caries and linear hypoplasia (short communication). Caries Res 1992; 26(3):205-208. | Comment |
| 1. Matee MI, Mikx FH, Maselle SY, van Palenstein Helderman WH. Mutans streptococci and lactobacilli in breast-fed children with rampant caries. Caries Res 1992; 26(3):183-187. | Different etiology |
| 1. Maturo RA, Cullen C. Dentistry for infants. J Mich Dent Assoc 1993; 75(6):30-5, 72. | Editorial |
| 1. Maupome G, Karanja N, Ritenbaugh C, Lutz T, Aickin M, Becker T. Dental caries in American Indian toddlers after a community-based beverage intervention. Ethn Dis 2010; 20(4):444-450. | Health promotion |
| 1. Maxim D, Danila I, Balcos C. [Clinical and therapeutic aspects of early childhood caries and severe early childhood caries--clinical cases]. Rev Med Chir Soc Med Nat Iasi 2011; 115(1):223-226. | Case report |
| 1. McCauley E, Mackie A. Breast milk activity during early lactation following maternal Tc-99(m) macroaggregated albumin lung perfusion scan. British Journal of Radiology 2002; 75(893):464-466. | Title not interest |
| 1. McClelland BR, McClelland PT. Pileated woodpecker nest and roost trees in Montana: links with old-growth and forest "health". Wildlife Society Bulletin 1999; 27(3):846-857. | Title not interest |
| 1. McIntosh EA, Wu AS, Buhler PL. Survey of dentists in the Ottawa-Carleton region concerning nursing bottle syndrome. Can J Public Health 1991; 82(5):349-350. | Title not interest |
| 1. McMaster P, McMaster HJ, Southall DP. Personal child health record and advice booklet programme in Tuzla, Bosnia Herzegovina. J R Soc Med 1996; 89(4):202-204. | Title not interest |
| 1. Menghini G, Steiner M, Imfeld T. [Early childhood caries--facts and prevention]. Ther Umsch 2008; 65(2):75-82. | Review |
| 1. Meon R. Unilateral rampant caries: an unusual presentation. J Clin Pediatr Dent 1991; 16(1):10-12. | Case report |
| 1. Messer LB. "Well baby" visits vital. Aust Dent Pract 1991; 2(6):23. | Letter |
| 1. Michal BC. "Bottle-mouth" caries. J La Dent Assoc 1969; 27(2):10-13. | Letter |
| 1. Michal BC. "Bottle-mouth" caries. 2. J Mercer Dent Soc 1970; 24(7):12. | Letter |
| 1. Milaat WA, Ghabrah TM. Health Profile of Balhareth area in Taif Region. J Family Community Med 1996; 3(1):39-47. | Title not interest |
| 1. Miller J, Vaughan-Williams E, Furlong R, Khosla T. Dental caries and children's weight. Lancet 1980; 2(8199):853. | Letter |
| 1. Milnes AR, Rubin CW, Karpa M, Tate R. A retrospective analysis of the costs associated with the treatment of nursing caries in a remote Canadian aboriginal preschool population. Community Dent Oral Epidemiol 1993; 21(5):253-260. | Title not interest |
| 1. Milnes AR. Description and epidemiology of nursing caries. J Public Health Dent 1996; 56(1):38-50. | Review |
| 1. Misra S, Tahmassebi JF, Brosnan M. Early childhood caries--a review. Dent Update 2007; 34(9):556-2, 564. | Review |
| 1. Moffatt ME. Nutritional problems of native canadian mothers and children. Can Fam Physician 1989; 35:377-382. | Title not interest |
| 1. Moreno Gonzalez JP, Barberia LE, Alexandrov PN, Morante Vadillo MV. [The importance of pediatrics in obtaining oral health in the child]. An Esp Pediatr 1983; 19(6):495-499. | Title not interest |
| 1. Morris RE, Gillespie G, Dashti A, Gopalakrishnan NS, al-Za'abi F. Early childhood caries in Kuwait: review and policy recommendations. East Mediterr Health J 1999; 5(5):1014-1022. | Review |
| 1. Morrison ML, Raphael MG. Modeling the Dynamics of Snags. Ecological Applications 1993; 3(2):322-330. | Title not interest |
| 1. Moss JP, Picton DC. The problems of dental development among the children on a Greek island. Dent Pract Dent Rec 1968; 18(12):442-448. | Title not interest |
| 1. Moss SJ. Preventive techniques in infant dental care. Nurse Pract 1988; 13(7):37-8, 40, 45, passim. | Title not interest |
| 1. Moss SJ. A cavity-free generation. J Dent Res 1991; 70(2):158. | Editorial |
| 1. . Mpouga-Paraskakes A. [Prolongued use of feeding bottle and type of caries. 150 cases]. Hell Stomatol Chron 1973; 17(1):9-13. | Case report |
| 1. Muller-Giamarchi M, Jasmin JR. [Baby-bottle syndrome]. Pediatrie 1990; 45(7-8):485-489. | Case report |
| 1. Murakami A, Furukawa I, Miyamoto S, Tanaka T, Ohigashi H. Curcumin combined with turmerones, essential oil components of turmeric, abolishes inflammation-associated mouse colon carcinogenesis. Biofactors 2013; 39(2):221-232. | Title not interest |
| 1. Naidu R, Nunn J, Forde M. Oral healthcare of preschool children in Trinidad: a qualitative study of parents and caregivers. Bmc Oral Health 2012; 12:27. | Title not interest |
| 1. Nainar SM, Mohummed S. Diet counseling during the infant oral health visit. Pediatr Dent 2004; 26(5):459-462. | Review |
| 1. Navia JM. Caries prevention in infants and young children: which etiologic factors should we address? J Public Health Dent 1994; 54(4):195-196. | Editorial |
| 1. Nelson M. Childhood nutrition and poverty. Proc Nutr Soc 2000; 59(2):307-315. | Different outcome |
| 1. Nielsen ST, Matheson I, Rasmussen JN, Skinnemoen K, Andrew E, Hafsahl G. Excretion of iohexol and metrizoate in human breast milk. Acta Radiol 1987; 28(5):523-526. | Title not interest |
| 1. Nussbaum BL. Was Don Quixote a dentist? ASDC J Dent Child 1993; 60(4):414. | Editorial |
| 1. O'Malley B, Brown AC, Tate M, Hertzler AA, Rojas MH. Infant feeding practices of migrant farm laborers in northern Colorado. J Am Diet Assoc 1991; 91(9):1084-1087. | Different outcome |
| 1. Oliveira AF, Chaves AM, Rosenblatt A. The influence of enamel defects on the development of early childhood caries in a population with low socioeconomic status: a longitudinal study. Caries Res 2006; 40(4):296-302. | Different etiology |
| 1. Omalley B, Brown AC, Tate M, Hertzler AA, Rojas MH. Infant-Feeding Practices of Migrant Farm Laborers in Northern Colorado. Journal of the American Dietetic Association 1991; 91(9):1084-1087. | Different outcome |
| 1. Opiyo N, English M. In-service training for health professionals to improve care of the seriously ill newborn or child in low and middle-income countries (Review). Cochrane Database of Systematic Reviews 2010. | Different outcome |
| 1. Ostos Garrido MJ, Gonzalez RE, Manrique MC. [Nursing caries. Therapeutic needs]. Av Odontoestomatol 1991; 7(3):197-203. | Title not interest |
| 1. Park BZ, Kinney MB, Steffensen JE. Putting teeth into your physical exam: Part 1. Children and adolesccents. J Fam Pract 1992; 35(4):459-462. | Review |
| 1. Paterson JE, Gao W, Sundborn G, Cartwright S. Maternal self-report of oral health in six-year-old Pacific children from South Auckland, New Zealand. Community Dent Oral Epidemiol 2011; 39(1):19-28. | Title not interest |
| 1. Pattanaporn K, Saraithong P, Khongkhunthian S, Aleksejuniene J, Laohapensang P, Chhun N et al. Mode of delivery, mutans streptococci colonization, and early childhood caries in three- to five-year-old Thai children. Community Dent Oral Epidemiol 2013; 41(3):212-223. | Different etiology |
| 1. Paunio P, Hakkinen P, Tenovuo J, Niva A, Lumikari M. Dip-slide scores of mutans streptococci and lactobacilli of Finnish mothers in the Turku area, Finland, during the first nursing year. Proc Finn Dent Soc 1988; 84(5-6):271-277. | Title not interest |
| 1. Pavlov MI, Naulin-Ifi C. [Plea for prevention and early management of baby bottle tooth decay syndrome]. Arch Pediatr 1999; 6(2):218-222. | Title not interest |
| 1. Pedro-Gil J, Lopez Andreu JA, Lono J, Nieto A, Lazaro C, Langa MN et al. [Morbidity, physical growth and psychopathology in marginal populations in suburban areas]. An Esp Pediatr 1992; 36 Suppl 48:118-123. | Title not interest |
| 1. Pendrys DG, Morse DE. Fluoride Supplement Use by Children in Fluoridated Communities. Journal of Public Health Dentistry 1995; 55(3):160-164. | Title not interest |
| 1. Peres KG, De Oliveira Latorre MR, Sheiham A, Peres MA, Victora CG, Barros FC. Social and biological early life influences on the prevalence of open bite in Brazilian 6-year-olds. Int J Paediatr Dent 2007; 17(1):41-49. | Different outcome |
| 1. Peretz B, Gleicher H, Gazit D, Eidelman E. Early root resorption of maxillary primary first molars in a child with severe congenital heart disease. J Clin Pediatr Dent 1997; 21(2):163-166. | Case reports |
| 1. Peretz B, Kafka I. Baby bottle tooth decay and complications during pregnancy and delivery. Pediatr Dent 1997; 19(1):34-36. | Different etiology |
| 1. Peretz B, Faibis S, Ever-Hadani P, Eidelman E. Children with baby bottle tooth decay treated under general anesthesia or sedation: behavior in a follow-up visit. J Clin Pediatr Dent 2000; 24(2):97-101. | Treatment |
| 1. Peretz B, Faibis S, Ever-Hadani P, Eidelman E. Dental health behavior of children with BBTD treated using general anesthesia or sedation, and of their parents in a recall examination. ASDC J Dent Child 2000; 67(1):50-4, 9. | Treatment |
| 1. Peretz B. Early childhood caries (ECC)/baby-bottle tooth decay--a reminder. Refuat Hapeh Vehashinayim 2002; 19(4):92. | Editorial |
| 1. Peretz B, Ram D, Azo E, Efrat Y. Preschool caries as an indicator of future caries: a longitudinal study. Pediatr Dent 2003; 25(2):114-118. | Different etiology |
| 1. Peretz B, Gluck G. Early childhood caries (ECC): a preventive-conservative treatment mode during a 12-month period. J Clin Pediatr Dent 2006; 30(3):191-194. | Treatment |
| 1. Persaud D, Bedri A, Ziemniak C, Moorthy A, Gudetta B, Abashawl A et al. Slower clearance of nevirapine resistant virus in infants failing extended nevirapine prophylaxis for prevention of mother-to-child HIV transmission. AIDS research and human retroviruses 2011; 27:823-829. | Title not interest |
| 1. Peters R. Risk factors in the nursing caries syndrome: a literature survey. J Dent Assoc S Afr 1994; 49(4):169-175. | Review |
| 1. Peterson A, Chandler S. ILCA's inside track: Take care of your breastfed baby's teeth. J Hum Lact 2008; 24(2):219-220. | Patient’s educational hand out |
| 1. Pietschnig B, Haschke F, Fried R, Schilling R, Thun-Hohenstein L, Heil M et al. [Preventive use of vitamin D and fluoride in Austria]. Wien Klin Wochenschr 1988; 100(19):646-648. | Title not interest |
| 1. Pistolas PJ. Growth and development in the pediatric patient. Funct Orthod 2004; 22(1):12-22. | Title not interest |
| 1. Platt MJ. Child health statistical review, 1997. Arch Dis Child 1997; 77(6):542-548. | Title not interest |
| 1. Plonka KA, Pukallus ML, Barnett AG, Walsh LJ, Holcombe TH, Seow WK. Mutans streptococci and lactobacilli colonization in predentate children from the neonatal period to seven months of age. Caries Res 2012; 46(3):213-220. | Title not interest |
| 1. Plonka KA, Pukallus ML, Barnett A, Holcombe TF, Walsh LJ, Seow WK. A controlled, longitudinal study of home visits compared to telephone contacts to prevent early childhood caries. Int J Paediatr Dent 2013; 23(1):23-31. | Health promotion |
| 1. Plotzitza B, Kneist S, Berger J, Hetzer G. Efficacy of chlorhexidine varnish applications in the prevention of early childhood caries. Eur J Paediatr Dent 2005; 6(3):149-154. | Title not interest |
| 1. Prince JR, Rose MR. Measurement of radioactivity in breast milk following (99m) Tc-Leukoscan injection. Nuclear Medicine Communications 2004; 25(9):963-966. | Title not interest |
| 1. Prowse TL, Saunders SR, Schwarcz HP, Garnsey P, Macchiarelli R, Bondioli L. Isotopic and dental evidence for infant and young child feeding practices in an imperial Roman skeletal sample. Am J Phys Anthropol 2008; 137(3):294-308. | Title not interest |
| 1. Qian H, Li C, Yue J. [Relationship between Streptococcus mutans, Lactobacillus spp. and lactate-producing level and nursing bottle caries]. Hua Xi Kou Qiang Yi Xue Za Zhi 2001; 19(6):369-371. | Title not interest |
| 1. Quartey J, Seidel S. Nursing caries and fluoride varnish. Tex Dent J 1998; 115(1):14-17. | Review |
| 1. Quinonez R, Santos RG, Wilson S, Cross H. The relationship between child temperament and early childhood caries. Pediatr Dent 2001; 23(1):5-10. | Different etiology |
| 1. Quinonez RB, Pahel BT, Rozier RG, Stearns SC. Follow-up preventive dental visits for Medicaid-enrolled children in the medical office. J Public Health Dent 2008; 68(3):131-138. | Title not interest |
| 1. Rajshekar SA, Laxminarayan N. Comparison of primary dentition caries experience in pre-term low birth-weight and full-term normal birth-weight children aged one to six years. J Indian Soc Pedod Prev Dent 2011; 29(2):128-134. | Different etiology |
| 1. Ramalingam L, Messer LB. Early childhood caries: an update. Singapore Dent J 2004; 26(1):21-29. | Review |
| 1. Ramires-Romito AC, Wanderley MT, Oliveira MD, Imparato JC, Correa MS. Biologic restoration of primary anterior teeth. Quintessence Int 2000; 31(6):405-411. | Case report |
| 1. Ramos-Gomez FJ, Weintraub JA, Gansky SA, Hoover CI, Featherstone JD. Bacterial, behavioral and environmental factors associated with early childhood caries. J Clin Pediatr Dent 2002; 26(2):165-173. | Different etiology |
| 1. Randell DM, Harth S, Seow WK. Preventive dental health practices of non-institutionalized Down syndrome children: a controlled study. J Clin Pediatr Dent 1992; 16(3):225-229. | Title not interest |
| 1. Redmo Emanuelsson IM, Wang XM. Demonstration of identical strains of mutans streptococci within Chinese families by genotyping. Eur J Oral Sci 1998; 106(3):788-794. | Title not interest |
| 1. Reisine S, Litt M. Social and psychological theories and their use for dental practice. Int Dent J 1993; 43(3 Suppl 1):279-287. | Title not interest |
| 1. Reisine S, Litt M, Tinanoff N. A biopsychosocial model to predict caries in preschool children. Pediatr Dent 1994; 16(6):413-418. | Different etiology |
| 1. Reisine S, Douglass JM. Psychosocial and behavioral issues in early childhood caries. Community Dent Oral Epidemiol 1998; 26(1 Suppl):32-44. | Review |
| 1. Reisine ST, Psoter W. Socioeconomic status and selected behavioral determinants as risk factors for dental caries. J Dent Educ 2001; 65(10):1009-1016. | Review |
| 1. Renfrew MJ, McCormick FM, Wade A, Quinn B, Dowswell T. Support for healthy breastfeeding mothers with healthy term babies. Cochrane Database of Systematic Reviews 2012. | Different outcome |
| 1. Rhyne RL, Hertzman PA. Pursuing community-oriented primary care in a Russian closed nuclear city: the Sarov-Los Alamos community health partnership. Am J Public Health 2002; 92(11):1740-1742. | Title not interest |
| 1. Ribeiro NM, Ribeiro MA. [Breastfeeding and early childhood caries: a critical review]. J Pediatr (Rio J ) 2004; 80(5 Suppl):S199-S210. | Review |
| 1. Richardson BD, Cleaton-Jones PE. Answer to nursing bottle syndrome problem. J Can Dent Assoc 1983; 49(1):11-12. | Letter |
| 1. Rigilano JC, Friedler EM, Ehemann LJ. Fluoride prescribing patterns among primary care physicians. J Fam Pract 1985; 21(5):381-385. | Title not interest |
| 1. Riley P, Lamont T. Triclosan/copolymer containing toothpastes for oral health. Cochrane Database of Systematic Reviews 2013. | Title not interest |
| 1. Riordan PJ. Dental fluorosis, dental caries and fluoride exposure among 7-year-olds. Caries Res 1993; 27(1):71-77. | Different etiology |
| 1. Ripa LW. Nursing habits and dental decay in infants: "nursing bottle caries". ASDC J Dent Child 1978; 45(4):274-275. | Title not interest |
| 1. Ripa LW. Nursing caries: a comprehensive review. Pediatr Dent 1988; 10(4):268-282. | Review |
| 1. Roberge JM, Desrochers A. Comparison of large snag characteristics between a primeval and a managed fir forest on the Gaspe Peninsula, Quebec. Canadian Journal of Forest Research-Revue Canadienne de Recherche Forestiere 2004; 34(11):2382-2386. | Title not interest |
| 1. Rubow S, Klopper J, Wasserman H, Baard B, van NM. The excretion of radiopharmaceuticals in human breast milk: additional data and dosimetry. Eur J Nucl Med 1994; 21(2):144-153. | Title not interest |
| 1. Rugg-Gunn AJ, Al-Mohammadi SM, Butler TJ. Malnutrition and developmental defects of enamel in 2- to 6-year-old Saudi boys. Caries Res 1998; 32(3):181-192. | Title not interest |
| 1. Rule JT. Recognition of dental caries. Pediatr Clin North Am 1982; 29(3):439-456 | Review |
| 1. Sakashita R, Inoue N, Kamegai T. Can oral health promotion help develop masticatory function and prevent dental caries? Community Dental Health 2006; 23(2):107-115. | Title not interest |
| 1. Salma S. Dentition and dental health. Nurs J India 2000; 91(5):102-104. | Title not interest |
| 1. Salmon TG, Jr. Early childhood caries: a private practitioner's perspective. Pediatr Dent 1997; 19(1):63-64. | Case report |
| 1. Salone LR, Vann WF, Jr., Dee DL. Breastfeeding: an overview of oral and general health benefits. J Am Dent Assoc 2013; 144(2):143-151. | Review |
| 1. Sanders BJ, Shapiro A, McKown CG. The case of good luck. Pediatr Dent 1994; 16(6):402. | Case report |
| 1. Santini A. The effective management of caries. Prim Dent J 2013; 2(3):5. | Editorial |
| 1. Saraiva MC, Bettiol H, Barbieri MA, Silva AA. Are intrauterine growth restriction and preterm birth associated with dental caries? Community Dent Oral Epidemiol 2007; 35(5):364-376. | Title not interest |
| 1. Sayyed T, Kandil M, Bashir O, Alnaser H. The relationship between term pre-eclampsia and the risk of early childhood caries. J Matern Fetal Neonatal Med 2014; 27(1):62-65. | Different etiology |
| 1. Scanlan D. Nursing bottle caries--a hospital program. Can Dent Hyg 1979; 13(4):86-87. | Case report |
| 1. Schalka MM, Rodrigues CR. [The importance of the pediatrician in oral health care promotion]. Rev Saude Publica 1996; 30(2):179-186. | Health promotion |
| 1. Schluter PJ, Durward C, Cartwright S, Paterson J. Maternal self-report of oral health in 4-year-old Pacific children from South Auckland, New Zealand: findings from the Pacific Islands Families Study. J Public Health Dent 2007; 67(2):69-77. | Title not interest |
| 1. Schroth RJ, Brothwell DJ, Moffatt ME. Caregiver knowledge and attitudes of preschool oral health and early childhood caries (ECC). Int J Circumpolar Health 2007; 66(2):153-167. | Health promotion |
| 1. Schuman NJ, Mills JA. A strategy for decreasing the incidence of baby bottle syndrome. J Tenn Dent Assoc 1981; 61(1):22-24. | Title not interest |
| 1. Schwartz SS, Rosivack RG, Michelotti P. A child's sleeping habit as a cause of nursing caries. ASDC J Dent Child 1993; 60(1):22-25. | Different etiology |
| 1. Schwarz D, Kuhne P, Dominok B, Melde S. [Alimentary intake of fluorides of artificially fed infants and effect on enamel tissue]. Z Gesamte Hyg 1990; 36(12):646-648. | Title not interest |
| 1. Shantinath SD, Breiger D, Williams BJ, Hasazi JE. The relationship of sleep problems and sleep-associated feeding to nursing caries. Pediatr Dent 1996; 18(5):375-378. | Different outcome |
| 1. Shaw LMA. Anthropology of the menopause. Research Papers in Fertility and Reproductive Medicine 2004; 1271:396-399. | Title not interest |
| 1. Sheehy E, Hirayama K, Tsamtsouris A. A survey of parents whose children had full-mouth rehabilitation under general anesthesia regarding subsequent preventive dental care. Pediatr Dent 1994; 16(5):362-364. | Treatment |
| 1. Sheikh C, Erickson PR. Evaluation of plaque pH changes following oral rinse with eight infant formulas. Pediatr Dent 1996; 18(3):200-204. | Title not interest |
| 1. Shein B, Tsamtsouris A, Rovero J. Self reported compliance and the effectiveness of prenatal dental education. J Clin Pediatr Dent 1991; 15(2):102-108. | Health promotion |
| 1. Sheller B, Williams BJ, Lombardi SM. Diagnosis and treatment of dental caries-related emergencies in a children's hospital. Pediatr Dent 1997; 19(8):470-475. | Treatment |
| 1. Sheller B, Williams BJ, Hays K, Mancl L. Reasons for repeat dental treatment under general anesthesia for the healthy child. Pediatr Dent 2003; 25(6):546-552. | Treatment |
| 1. Silberman SL, Trubman A, Duncan WK, Meydrech EF. Prevalence of primary canine hypoplasia of the mandibular teeth. Pediatr Dent 1991; 13(6):356-360. | Title not interest |
| 1. Silver DH. Improvements in the dental health of 3-year-old Hertfordshire children after 8 years. The relationship to social class. Br Dent J 1982; 153(5):179-183. | Title not interest |
| 1. Simpson TC, Needleman I, Wild SH, Moles DR, Mills EJ. Treatment of periodontal disease for glycaemic control in people with diabetes. Cochrane Database of Systematic Reviews 2010. | Title not interest |
| 1. Simpson WJ, Tuba J. An investigation of fluoride concentration in the milk of nursing mothers. J Oral Med 1968; 23(3):104-106. | Title not interest |
| 1. Skeie MS, Skaret E, Espelid I, Misvaer N. Do public health nurses in Norway promote information on oral health? Bmc Oral Health 2011; 11:23. | Health promotion |
| 1. Slavkin HC. Streptococcus mutans, early childhood caries and new opportunities. J Am Dent Assoc 1999; 130(12):1787-1792. | Different etiology |
| 1. Smith TJ, Phipps AW, Fell TP, Harrison JD. Transfer of alkaline earth elements in mothers' milk and doses from 45Ca, 90Sr and 226Ra. Radiat Prot Dosimetry 2003; 105(1-4):273-277. | Title not interest |
| 1. Sonis A, Castle J, Duggan C. Infant nutrition: implication for somatic growth, adult onset diseases, and oral health. Curr Opin Pediatr 1997; 9(3):289-297. | Title not interest |
| 1. Sorin MS. Cockayne's syndrome: dental findings and management. J Clin Pediatr Dent 1994; 18(4):299-302. | Title not interest |
| 1. Sperry NJ. Should dental health professionals encourage the consumption of milk? Dent Hyg (Chic ) 1983; 57(1):23-27. | Different outcome |
| 1. Staehle HJ. [Therapeutic possibilities in dental care of small children with bottle caries syndrome]. Quintessenz 1989; 40(3):423-443. | Treatment |
| 1. Stevens A, Freeman R. The role of the mother-child interaction as a factor in nursing caries (ECC): a preliminary communication. Eur J Paediatr Dent 2004; 5(2):81-85. | Different etiology |
| 1. Sturt AS, Dokubo EK, Sint TT. Antiretroviral therapy (ART) for treating HIV infection in ART-eligible pregnant women. Cochrane Database of Systematic Reviews 2010. | Title not interest |
| 1. Syahrial D, Abdul-Kadir R, Yassin Z, Jali NM. Knowledge and attitudes of parents of children with nursing bottle syndrome in Serdang, Malaysia. J Nihon Univ Sch Dent 1995; 37(3):146-151. | Treatment |
| 1. Szczepanska J, Sakowska D. [Quantitative and qualitative characteristics of the bacterial flora of saliva and dental plaque in artificially fed children]. Czas Stomatol 1990; 43(11-12):666-671. | Title not interest |
| 1. Taatz H. [Orthodontic prophylaxis]. Stomatol DDR 1978; 28(5):342-351. | Title not interest |
| 1. Taipale T, Pienihakkinen K, Salminen S, Jokela J, Soderling E. Bifidobacterium animalis subsp. lactis BB-12 administration in early childhood: a randomized clinical trial of effects on oral colonization by mutans streptococci and the probiotic. Caries Res 2012; 46(1):69-77. | Title not interest |
| 1. Takeda Y, Horiuchi N, Nakata M. [An odontological study on Down's syndrome. Part 3: Dental caries of the deciduous teeth]. Shoni Shikagaku Zasshi 1989; 27(1):85-91. | Title not interest |
| 1. Tankkunnasombut S, Youcharoen K, Wisuttisak W, Vichayanrat S, Tiranathanagul S. Early colonization of mutans streptococci in 2- to 36-month-old Thai children. Pediatr Dent 2009; 31(1):47-51. | Title not interest |
| 1. Targino AG, Rosenblatt A, Oliveira AF, Chaves AM, Santos VE. The relationship of enamel defects and caries: a cohort study. Oral Dis 2011; 17(4):420-426. | Different etiology |
| 1. Tedstone A, Dunce N, Aviles M, Shetty P, Daniels L. Effectiveness of interventions to promote healthy feeding in infants under one year of age (Structured abstract). Database of Abstracts of Reviews of Effects 1998;80. | Health promotion |
| 1. Teixeira AKM, de Menezes LMB, Dias AA, de Alencar CHM, de Almeida MEL. Analysis of protection or risk factors for dental fluorosis in 6 to 8 year-old children in Fortaleza, Brazil. Revista Panamericana de Salud Publica-Pan American Journal of Public Health 2010; 28(6):421-428. | Different outcome |
| 1. Terrell ML, Manatunga AK, Small CM, Cameron LL, Wirth J, Blanck HM et al. A decay model for assessing polybrominated biphenyl exposure among women in the Michigan Long-Term PBB Study. J Expo Sci Environ Epidemiol 2008; 18(4):410-420. | Title not interest |
| 1. Tewari A, Gauba K, Goyal A. Evaluation of the change in the knowledge of community regarding infant dental care subsequent to intervention strategies through existing health manpower in rural areas of Haryana (India). J Indian Soc Pedod Prev Dent 1994; 12(1):29-34. | Health promotion |
| 1. Thaver D, Saeed MA, Bhutta ZA. Pyridoxine (vitamin B6) supplementation in pregnancy. Cochrane Database of Systematic Reviews 2006. | Title not interest |
| 1. Thomson ME, Thomson CW, Chandler NP. In vitro and intra-oral investigations into the cariogenic potential of human milk. Caries Res 1996; 30(6):434-438. | In vitro study |
| 1. Thorild I, Lindau-Jonson B, Twetman S. Prevalence of salivary Streptococcus mutans in mothers and in their preschool children. Int J Paediatr Dent 2002; 12(1):2-7. | Title not interest |
| 1. Thorstrom R, Lind J. First nest description, breeding, ranging and foraging behaviour of the Short-legged Ground Roller Brachypteracias leptosomus in Madagascar. Ibis 1999; 141(4):569-576. | Title not interest |
| 1. Tinanoff N. The Early Childhood Caries Conference--October 18-19, 1997. Pediatr Dent 1997; 19(8):453-454. | Title not interest |
| 1. Tinanoff N. Introduction to the Early Childhood Caries Conference: initial description and current understanding. Community Dent Oral Epidemiol 1998; 26(1 Suppl):5-7. | Review |
| 1. Tinanoff N, Daley NS, O'Sullivan DM, Douglass JM. Failure of intense preventive efforts to arrest early childhood and rampant caries: three case reports. Pediatr Dent 1999; 21(3):160-163. | Health promotion |
| 1. Ton Q, Frenkel L. HIV drug resistance in mothers and infants following use of antiretrovirals to prevent mother-to-child transmission. Curr HIV Res 2013; 11(2):126-136. | Title not interest |
| 1. Tong L, Geng FZ, Liu SJ. [A study of oral colonization of mutans streptococci and feeding habits in infants]. Hua Xi Kou Qiang Yi Xue Za Zhi 2004; 22(1):43-45. | Title not interest |
| 1. Trask PA. Hazards of the baby bottle. J Am Dent Assoc 1982; 104(1):13. | Letter |
| 1. Truhe T. Diet and caries. Dent Today 1996; 15(9):60, 62-60, 65. | Review |
| 1. Tuli A, Singh A. Early childhood caries and oral rehabilitation. A treatment quandary. Eur J Paediatr Dent 2010; 11(4):181-184. | Treatment |
| 1. Updyke JR. Use of the sippy cup. Pediatr Dent 2002; 24(2):97. | Letter |
| 1. Vachirarojpisan T, Shinada K, Kawaguchi Y. The process and outcome of a programme for preventing early childhood caries in Thailand. Community Dent Health 2005; 22(4):253-259. | Title not interest |
| 1. Vadiakas G. Case definition, aetiology and risk assessment of early childhood caries (ECC): a revisited review. Eur Arch Paediatr Dent 2008; 9(3):114-125. | Review |
| 1. Vadiakas GP, Oulis C. A review of dentine-bonding agents and an account of clinical applications in paediatric dentistry. Int J Paediatr Dent 1994; 4(4):209-216. | Title not interest |
| 1. Van den Steen E, Bottenberg P. [Removable prosthesis in a 4-year-old child with nursing bottle caries and possible ectodermal dysplasia]. Rev Belge Med Dent (1984 ) 2004; 59(2):89-93. | Case reports |
| 1. van der Sanden-Stoelinga MS, Koelen MA, Hielkema-de Meij JE. The making of a nation-wide campaign fighting the nursing caries. Int J Dent Hyg 2003; 1(1):16-22. | Health promotion |
| 1. van ET, Eijkman MA, Hoogstraten J. Parents and nursing-bottle caries. ASDC J Dent Child 1996; 63(4):271-274. | Title not interest |
| 1. van HJ, Gibbs G, Butera C. Oral flora of children with "nursing bottle caries". J Dent Res 1982; 61(2):382-385. | Different etiology |
| 1. Vann WF, Jr., Lee JY, Baker D, Divaris K. Oral health literacy among female caregivers: impact on oral health outcomes in early childhood. J Dent Res 2010; 89(12):1395-1400. | Life quality |
| 1. Vazquez-Nava F, Vazquez-Rodriguez EM, Saldivar-Gonzalez AH, Lin-Ochoa D, Martinez-Perales GM, Joffre-Velazquez VM. Association between obesity and dental caries in a group of preschool children in Mexico. J Public Health Dent 2010; 70(2):124-130. | Different outcome |
| 1. Vestman NR, Timby N, Holgerson PL, Kressirer CA, Claesson R, Domellof M et al. Characterization and in vitro properties of oral lactobacilli in breastfed infants. Bmc Microbiology 2013; 13. | Title not interest |
| 1. Vichayanrat T, Steckler A, Tanasugarn C, Lexomboon D. The evaluation of a multi-level oral health intervention to improve oral health practices among caregivers of preschool children. Southeast Asian J Trop Med Public Health 2012; 43(2):526-539. | Title not interest |
| 1. Villa AE, Guerrero S, Icaza G, Villalobos J, Anabalon M. Dental fluorosis in Chilean children: evaluation of risk factors. Community Dent Oral Epidemiol 1998; 26(5):310-315. | Different etiology |
| 1. Vitolo MR, Bortolini GA, Feldens CA, Drachler ML. [Impacts of the 10 Steps to Healthy Feeding in Infants: a randomized field trial]. Cad Saude Publica 2005; 21(5):1448-1457. | Title not interest |
| 1. Wadhawan S, Kumar JV, Badner VM, Green EL. Early childhood caries-related visits to hospitals for ambulatory surgery in New York State. J Public Health Dent 2003; 63(1):47-51. | Treatment |
| 1. Wakaguri S, Aida J, Osaka K, Morita M, Ando Y. Association between caregiver behaviours to prevent vertical transmission and dental caries in their 3-year-old children. Caries Res 2011; 45(3):281-286. | Title not interest |
| 1. Waldman HB. Twenty-five years of increasing use of pediatric dental services. ASDC J Dent Child 1993; 60(4):399-402. | Health promotion |
| 1. Waldman HB, Perlman SP. Are we reaching very young children with needed dental services? ASDC J Dent Child 1999; 66(6):390-4, 366. | Title not interest |
| 1. Walker C, White VA. Home visits providing diet advice may reduce early childhood caries. Do home visits that provide new mothers with advice about breastfeeding and weaning reduce the odds of infants developing early childhood caries? Evid Based Dent 2007; 8(4):108-109. | Comment |
| 1. Walker RB, Conn JA, Davies MJ, Moore VM. Mothers' views on feeding infants around the time of weaning. Public Health Nutr 2006; 9(6):707-713. | Title not interest |
| 1. Walton J, Messer LB. Dental-Caries and Fluorosis in Breast-Fed Vs Formula-Fed Infants in A Fluoridated Community. Journal of Dental Research 1977; 56:B103. | Title not interest |
| 1. Wan AKL, Seow WK, Purdie DM, Bird PS, Walsh LJ, Tudehope DI. Oral colonization of Streptococcus mutans in six-month-old predentate infants. Journal of Dental Research 2001; 80(12):2060-2065. | Title not interest |
| 1. Wandera A. Anticipatory guidance in infant oral health. J Mich Dent Assoc 1998; 80(9):28, 55-28, 59. | Title not interest |
| 1. Wang HS, Cui HM, Peng X, Fang J, Zuo ZC, Liu J et al. Effects of High Dietary Fluorine on Fatty Acid Composition in Breast Muscle of Broilers. Fluoride 2012; 45(2):100-107. | Title not interest |
| 1. Watanabe S. Salivary clearance from different regions of the mouth in children. Caries Res 1992; 26(6):423-427. | Title not interest |
| 1. Weber G, Singhal RL, Prajda N, Yeh YA, Look KY, Sledge GW, Jr. Regulation of signal transduction. Adv Enzyme Regul 1995; 35:1-21. | Title not interest |
| 1. Weber G. The need for evidence based recommendations for the prevention of oral diseases. Int Dent J 2009; 59(3):168-169. | Letter |
| 1. Weinstein P, Domoto P, Wohlers K, Koday M. Mexican-American parents with children at risk for baby bottle tooth decay: pilot study at a migrant farmworkers clinic. ASDC J Dent Child 1992; 59(5):376-383. | Different outcome |
| 1. Weinstein P, Domoto P, Koday M, Leroux B. Results of a promising open trial to prevent baby bottle tooth decay: a fluoride varnish study. ASDC J Dent Child 1994; 61(5-6):338-341. | Health promotion |
| 1. Weinstein P, Oberg D, Domoto PK, Jeffcott E, Leroux B. A prospective study of the feeding and brushing practices of WIC mothers: six- and twelve-month data and ethnicity and familial variables. ASDC J Dent Child 1996; 63(2):113-117. | Different outcome |
| 1. Weinstein P, Troyer R, Jacobi D, Moccasin M. Dental experiences and parenting practices of Native American mothers and caretakers: what we can learn for the prevention of baby bottle tooth decay. ASDC J Dent Child 1999; 66(2):120-6, 85. | Title not interest |
| 1. Weinstein P, Riedy CA. The reliability and validity of the RAPIDD scale: readiness assessment of parents concerning infant dental decay. ASDC J Dent Child 2001; 68(2):129-35, 142. | Title not interest |
| 1. Wetzel WE, Lehn W, Grieb A. [Carotene jaundice in infants with "sugar nursing bottle syndrome"]. Monatsschr Kinderheilkd 1989; 137(10):659-661. | Title not interest |
| 1. Wetzel WE, Grieb A, Pabst W. [Extraction of the deciduous anterior teeth and its consequences in children with the nursing bottle syndrome]. Schweiz Monatsschr Zahnmed 1993; 103(3):269-275. | Different etiology |
| 1. Wetzel WE, Hanisch S, Sziegoleit A. [The germ colonization of the oral cavity in small children with the nursing bottle syndrome]. Schweiz Monatsschr Zahnmed 1993; 103(9):1107-1112. | Different etiology |
| 1. Weyers H. [Deciduous tooth caries in the early teeth and lengthy nursing]. Zahnarztl Mitt 1984; 74(15):1654-1657. | Letter |
| 1. White GE. Nutrition in the practice of pediatric dentistry. Dent Clin North Am 1976; 20(3):507-517. | Title not interest |
| 1. Wight NE. Management of common breastfeeding issues. Pediatr Clin North Am 2001; 48(2):321-344. | Letter |
| 1. Williams SA, Hargreaves JA. An inquiry into the effects of health related behaviour on dental health among young Asian children resident in a fluoridated city in Canada. Community Dent Health 1990; 7(4):413-420. | Title not interest |
| 1. Williams SA, Ahmed IA, Hussain P. Ethnicity, health and dental care--perspectives among British Asians: 1. Dent Update 1991; 18(4):154-161. | Title not interest |
| 1. Winterbottom JB, Smyth R, Jacoby A, Baker GA. Preconception counselling for women with epilepsy to reduce adverse pregnancy outcome. Cochrane Database of Systematic Reviews 2008. | Title not interest |
| 1. Wondwossen F, Astrom AN, Bjorvatn K, Bardsen A. Sociodemographic and behavioural correlates of severe dental fluorosis. International Journal of Paediatric Dentistry 2006; 16(2):95-103. | Title not interest |
| 1. Worthington H, V, Clarkson JE, Bryan G, Furness S, Glenny AM, Littlewood A et al. Interventions for preventing oral mucositis for patients with cancer receiving treatment. Cochrane Database of Systematic Reviews 2011. | Health promotion |
| 1. Wyne AH. Early childhood caries: nomenclature and case definition. Community Dent Oral Epidemiol 1999; 27(5):313-315. | Case report |
| 1. Yagot K, Nazhat NY, Kuder SA. Prolonged nursing-habit caries index. J Int Assoc Dent Child 1990; 20(1):8-10. | Different outcome |
| 1. Yakushiji T, Watanabe I, Kuwabara K, Tanaka R, Kashimoto T, Kunita N et al. Postnatal transfer of PCBs from exposed mothers to their babies: influence of breast-feeding. Arch Environ Health 1984; 39(5):368-375. | Different outcome |
| 1. Yamaguchi K, Holman DJ. Longitudinal analysis of permanent tooth emergence in Japanese children. Anthropological Science 2010; 118(2):141-149. | Title not interest |
| 1. Yengopal V, Harnekar SY, Patel N, Siegfried N. Dental fillings for the treatment of caries in the primary dentition. Cochrane Database of Systematic Reviews 2009. | Treatment |
| 1. Yiu CK, Wei SH. Management of rampant caries in children. Quintessence Int 1992; 23(3):159-168. | Review |
| 1. Young KL, Levy SM, Kuthy RA. Dietary fluoride supplements for Nebraska's children--the role of the physician. Nebr Med J 1989; 74(9):265-270. | Title not interest |
| 1. Zehetbauer S, Wojahn T, Hiller KA, Schmalz G, Ruhl S. Resemblance of salivary protein profiles between children with early childhood caries and caries-free controls. Eur J Oral Sci 2009; 117(4):369-373. | Different etiology |
| 1. Zenker KH. [Importance of breast feeding from the dentist's view]. Zahnarztl Mitt 1969; 59(23):1196-1197. | Letter |
| 1. Zero DT, Lussi A. Behavioral factors. Monogr Oral Sci 2006; 20:100-105. | Review |
| 1. Zervanos NJ. Dental care. J Fam Pract 1993; 36(3):263-264. | Comment |
| 1. Ziegler P, Briefel R, Clusen N, Devaney B. Feeding Infants and Toddlers Study (FITS): development of the FITS survey in comparison to other dietary survey methods. J Am Diet Assoc 2006; 106(1 Suppl 1):S12-S27. | Different outcome |
| 1. Patient's page. Keeping your child's teeth healthy begins early. J Okla Dent Assoc 2013; 104(7):8. | Patient’s hand out |
| 1. Khadra-Eid J, Baudet D, Fourny M, Sellier E, Brun C, Francois P. [Development of a screening scale for children at risk of baby bottle tooth decay]. Arch Pediatr 2012; 19(3):235-241. | Language other than English other than English |
| 1. Droz D. [Breastfeeding and the risk of dental caries]. Arch Pediatr 2003; 10 Suppl 1:9s-11s. | Language other than English other than English |
| 1. Mizoguchi K, Kurumado K, Tango T, Minowa M. [Study on factors for caries and infant feeding characteristics in children aged 1.5-3 years in a Kanto urban area]. Nihon Koshu Eisei Zasshi 2003; 50(9):867-878. | Language other than English other than English |
| 1. Beiruti N, Taifour M. [Prevalence of nursing caries among children 3-5 years old in Damascus]. East Mediterr Health J 2000; 6(2-3):500-506. | Language other than English other than English |
| 1. Creedon MI, O'Mullane DM. Factors affecting caries levels amongst 5-year-old children in County Kerry, Ireland. Community Dent Health 2001; 18(2):72-78. | Does not compare breastfeeding and bottle feeding. |
| 1. Livny A, Sgan-Cohen HD. A review of a community program aimed at preventing early childhood caries among Jerusalem infants--a brief communication. J Public Health Dent 2007; 67(2):78-82. | Review |
| 1. Tsubouchi J, Higashi T, Shimono T, Domoto PK, Weinstein P. A study of baby bottle tooth decay and risk factors for 18-month old infants in rural Japan. ASDC J Dent Child 1994; 61(4):293-298. | Does not compare breastfeeding and bottlefeeding |
| 1. Tsubouchi J, Tsubouchi M, Maynard RJ, Domoto PK, Weinstein P. A study of dental caries and risk factors among Native American infants. ASDC J Dent Child 1995; 62(4):283-287. | Descriptive study. |
| 1. Sinton J, Valaitis R, Passarelli C, Sheehan D, Hesch R. A systematic overview of the relationship between infant feeding caries and breast-feeding. Ont Dent 1998; 75(9):23-27. | Review about breastfeeding only. |
| 1. Neesanan N, Limpanich L.Pilot study in young Thai children with delayed bottle-weaning at Queen Sirikit National Institute of Child Health: does it affect iron status? Med Assoc Thai. 2014 Jun;97 Suppl 6:S189-94. | Only bottlefeeding. |
| 1. Peltzer K, Mongkolchati A, Satchaiyan G, Rajchagool S, Pimpak T. [Sociobehavioral factors associated with caries increment: a longitudinal study from 24 to 36 months old children in Thailand.](http://www.ncbi.nlm.nih.gov/pubmed/25329535) [Int J Environ Res Public Health.](http://www.ncbi.nlm.nih.gov/pubmed/25329535) 2014 Oct 17;11(10):10838-50. | Only bottlefeeding. |
| 1. Hong L, Levy SM, Warren JJ, Broffitt B. [Infant breast-feeding and childhood caries: a nine-year study.](http://www.ncbi.nlm.nih.gov/pubmed/25198001) Pediatr Dent. 2014 Jul-Aug;36(4):342-7. | Only breastfeeding. |
| 1. Al-Zahrani AM, Al-Mushayt AS, Otaibi MF, Wyne AH. Knowledge and attitude of Saudi mothers towards their preschool children's oral health. Pak J Med Sci. 2014 Jul;30(4):720-4. | Health promotion. |
| 1. Carvalho JC, Silva EF, Vieira EO, Pollaris A, Guillet A, Mestrinho HD. Oral Health Determinants and Caries Outcome among Non-Privileged Children. Caries Res. 2014 Jun 5;48(6):515-523. | Only bottle feeding. |
| 1. Tanaka K, Miyake Y. Low birth weight, preterm birth or small-for-gestational-age are not associated with dental caries in young Japanese children. BMC Oral Health. 2014 Apr 14;14:38. | Different etiology. |
| 1. Ribeiro CC, da Silva MC, Machado CM, Ribeiro MR, Thomaz EB. [Is the severity of caries associated with malnutrition in preschool children?]. Cien Saude Colet. 2014 Mar;19(3):957-65. | Different outcome: malnutrition. |
| 1. Kühnisch J, Mach D, Thiering E, Brockow I, Hoffmann U, Neumann C, Heinrich-Weltzien R, Bauer CP, Berdel D, von Berg A, Koletzko S, Garcia-Godoy F, Hickel R, Heinrich J; GINI Plus 10 Study Group. Respiratory diseases are associated with molar-incisor hypomineralizations. Swiss Dent J. 2014;124(3):286-93. | Different outcome: molar-incisor hypomineralizations. |
| 1. Chaffee BW, Feldens CA, Vítolo MR. Association of long-duration breastfeeding and dental caries estimated with marginal structural models. Ann Epidemiol. 2014 Jun;24(6):448-54. | Only breastfeeding. |
| 1. Begzati A, Bytyci A, Meqa K, Latifi-Xhemajli B, Berisha M. Mothers' behaviours and knowledge related to caries experience of their children. Oral Health Prev Dent. 2014;12(2):133-40. | Prevention and health promotion. |
| 1. Wang XT, Ge LH. [Influence of feeding patterns on the development of teeth, dentition and jaw in children]. [Beijing Da Xue Xue Bao.](http://www.ncbi.nlm.nih.gov/pubmed/25686355) 2015; 47(1):191-5. | Language other than English other than English. |
| 1. Martonffy AL. Oral health: prevention of dental disease. FP Essent. 2015; 428:11-5. | Letter. |
| 1. [Ajetunmobi OM](http://www.ncbi.nlm.nih.gov/pubmed/?term=Ajetunmobi OM%5BAuthor%5D&cauthor=true&cauthor_uid=25556021), [Whyte B](http://www.ncbi.nlm.nih.gov/pubmed/?term=Whyte B%5BAuthor%5D&cauthor=true&cauthor_uid=25556021), [Chalmers J](http://www.ncbi.nlm.nih.gov/pubmed/?term=Chalmers J%5BAuthor%5D&cauthor=true&cauthor_uid=25556021), [Tappin DM](http://www.ncbi.nlm.nih.gov/pubmed/?term=Tappin DM%5BAuthor%5D&cauthor=true&cauthor_uid=25556021), [Wolfson L](http://www.ncbi.nlm.nih.gov/pubmed/?term=Wolfson L%5BAuthor%5D&cauthor=true&cauthor_uid=25556021), [Fleming M](http://www.ncbi.nlm.nih.gov/pubmed/?term=Fleming M%5BAuthor%5D&cauthor=true&cauthor_uid=25556021), [MacDonald A](http://www.ncbi.nlm.nih.gov/pubmed/?term=MacDonald A%5BAuthor%5D&cauthor=true&cauthor_uid=25556021), [Wood R](http://www.ncbi.nlm.nih.gov/pubmed/?term=Wood R%5BAuthor%5D&cauthor=true&cauthor_uid=25556021), [Stockton DL](http://www.ncbi.nlm.nih.gov/pubmed/?term=Stockton DL%5BAuthor%5D&cauthor=true&cauthor_uid=25556021). Breastfeeding is associated with reduced childhood hospitalization: evidence from Scottish birth cohort (1997-2009). [J Pediatr.](http://www.ncbi.nlm.nih.gov/pubmed/25556021) 2015; 166(3):620-625. | Different outcome: hospitalization. |
| 1. Kowash MB. Severity of early childhood caries in preschool children attending Al-Ain Dental Centre, United Arab Emirates. [Eur Arch Paediatr Dent.](http://www.ncbi.nlm.nih.gov/pubmed/25526933) 2014 Dec 20. | Only bottle feeding. |
| 1. [Wagner Y](http://www.ncbi.nlm.nih.gov/pubmed/?term=Wagner Y%5BAuthor%5D&cauthor=true&cauthor_uid=24885353), [Heinrich-Weltzien R](http://www.ncbi.nlm.nih.gov/pubmed/?term=Heinrich-Weltzien R%5BAuthor%5D&cauthor=true&cauthor_uid=24885353). Pediatrians’ oral health recommendations from 0-to- 3-years-old children: results of a survey in Thuringya, Germany. [BMC Oral Health.](http://www.ncbi.nlm.nih.gov/pubmed/24885353) 2014 May 1;14:44. doi: 10.1186/1472-6831-14-44. | Title not interest. |
| 1. Zhang S, Liu J, Lo EC, Chu CH. Dental caries status of Bulang preschool children in Southwest China. [BMC Oral Health.](http://www.ncbi.nlm.nih.gov/pubmed/24593701) 2014 Mar 4;14:16. doi: 10.1186/1472-6831-14-16. | Only bottle feeding. |
| 1. [Congiu G](http://www.ncbi.nlm.nih.gov/pubmed/?term=Congiu G%5BAuthor%5D&cauthor=true&cauthor_uid=24117607), [Campus G](http://www.ncbi.nlm.nih.gov/pubmed/?term=Campus G%5BAuthor%5D&cauthor=true&cauthor_uid=24117607), [Sale S](http://www.ncbi.nlm.nih.gov/pubmed/?term=Sale S%5BAuthor%5D&cauthor=true&cauthor_uid=24117607), [Spano G](http://www.ncbi.nlm.nih.gov/pubmed/?term=Spano G%5BAuthor%5D&cauthor=true&cauthor_uid=24117607), [Cagetti MG](http://www.ncbi.nlm.nih.gov/pubmed/?term=Cagetti MG%5BAuthor%5D&cauthor=true&cauthor_uid=24117607), [Lugliè PF](http://www.ncbi.nlm.nih.gov/pubmed/?term=Lugliè PF%5BAuthor%5D&cauthor=true&cauthor_uid=24117607). Early childhood caries and associated determinants: a cross-sectional study on Italian preschool children. [J Public Health Dent.](http://www.ncbi.nlm.nih.gov/pubmed/24117607) 2014 Spring;74(2):147-52. doi: 10.1111/jphd.12038. | Does not compare breastfeeding and bottlefeeding. |
| 1. [Nakayama Y](http://www.ncbi.nlm.nih.gov/pubmed/?term=Nakayama Y%5BAuthor%5D&cauthor=true&cauthor_uid=25721070), [Mori M](http://www.ncbi.nlm.nih.gov/pubmed/?term=Mori M%5BAuthor%5D&cauthor=true&cauthor_uid=25721070). Association between nocturnal breastfeeding and snacking habits and the risk of early childhood caries in 18- to 23-month-old Japanese children. [J Epidemiol.](http://www.ncbi.nlm.nih.gov/pubmed/25721070) 2015 Feb 5;25(2):142-7. doi: 10.2188/jea.JE20140097. | Does not compare breastfeeding and bottlefeeding. |
| 1. Verrips GH, Frencken JE, Kalsbeek H, ter Horst G, Filedt Kok-Weimar TL. Risk indicators and potential risks for caries in 5-years-old of different ethnic groups in Amsterdam. Community Dent Oral Epidemiol. 1992; 20: 256-260. | Different etiology. |
