# Supplementary material for: Breast and Bottle Feeding as Risk Factors for Dental Caries: A Systematic Review and Meta-Analysis
Source: PLoS One. 2015 Nov 18;10(11):e0142922. doi: 10.1371/journal.pone.0142922 (PMC4651315; doi:10.1371/journal.pone.0142922)
Supplement: S2 Appendix — (DOC) [file pone.0142922.s003.doc]

| Reference  **S2 Appendix**. **List of all studies selected for full text analysis and reasons for exclusion.** | Classification |
| --- | --- |
| 1. Aminabadi NA, Ghoreishizadeh A, Ghoreishizadeh M, Oskouei SG, Ghojazadeh M. Can Child Temperament Be Related to Early Childhood Caries? Caries Research 2014; 48(1):3-12. | NOT SELECTED. Does not compare breastfeeding with bottle feeding. |
| 1. Correa-Faria P, Martins PA, Vieira-Andrade RG, Marques LS, Ramos-Jorge ML. Factors associated with the development of early childhood caries among Brazilian preschoolers. Brazilian Oral Research 2013; 27(4):356-362. | NOT SELECTED. Statistical data unavailable. |
| 1. Aimutis WR. Lactose cariogenicity with an emphasis on childhood dental caries. International Dairy Journal 2012; 22(2):152-158. | NOT SELECTED. Review |
| 1. Arora A, Scott JA, Bhole S, Do L, Schwarz E, Blinkhorn AS. Early childhood feeding practices and dental caries in preschool children: a multi-centre birth cohort study. Bmc Public Health 2011; 11. | NOT SELECTED. Study protocol |
| 1. Helderman WV, Soe W, van't Hof MA. Risk factors of early childhood caries in a southeast Asian population. Journal of Dental Research 2006; 85(1):85-88. | NOT SELECTED. Only breastfeeding. |
| 1. Azevedo TDPL, Bezerra ACB, de Toledo OA. Feeding habits and severe early childhood caries in Brazilian an preschool children. Pediatric Dentistry 2005; 27(1):28-33. | NOT SELECTED. Breastfeeding during night. |
| 1. Del Valle LL, Velazquez-Quintana Y, Weinstein P, Domoto P, Leroux B. Early childhood caries and risk factors in rural Puerto Rican children. Journal of Dentistry for Children 1998; 65(2):132-+. | NOT SELECTED. Only bottle feeding. |
| 1. Olojugba OO, Hardwick JL. Relationship of Breast-Feeding and Bottle-Feeding During Infancy to Caries Experience in Nigerian Children. Caries Research 1979; 13(2):101-102. | NOT SELECTED. Does not compare breastfeeding and bottle feeding. |
| 1. Nobile CG, Fortunato L, Bianco A, Pileggi C, Pavia M. Pattern and severity of early childhood caries in Southern Italy: a preschool-based cross-sectional study. Bmc Public Health 2014; 14(1):206. | NOT SELECTED. Statistical data unavailable. |
| 1. Prakasha SS, Vinit GB, Giri KY, Alam S. Feeding practices and early childhood caries: a cross-sectional study of preschool children in kanpur district, India. ISRN Dent 2013; 2013:275193. | NOT SELECTED. Statistical data unavailable. |
| 1. Nazar H, Al-Mutawa S, Ariga J, Soparkar P, Mascarenhas AK. Caries prevalence, oral hygiene, and oral health habits of kuwaiti infants and toddlers. Med Princ Pract 2014; 23:125-128. | NOT SELECTED. Only bottle feeding. |
| 1. Boka V, Trikaliotis A, Kotsanos N, Karagiannis V. Dental caries and oral health-related factors in a sample of Greek preschool children. Eur Arch Paediatr Dent 2013; 14(6):363-368. | NOT SELECTED. Only bottle feeding. |
| 1. Congiu G, Campus G, Sale S, Spano G, Cagetti MG, Luglie PF. Early childhood caries and associated determinants: a cross-sectional study on Italian preschool children. J Public Health Dent 2014(2):147-152. | NOT SELECTED. Only bottle feeding. |
| 1. Bissar A, Schiller P, Wolff A, Niekusch U, Schulte AG. Factors contributing to severe early childhood caries in south-west Germany. Clin Oral Investig 2014. | NOT SELECTED. Statistical data unavailable. |
| 1. Skrivele S, Care R, Berzina S, Kneist S, de Moura-Sieber V, de MR et al. Caries and its risk factors in young children in five different countries. Stomatologija 2013; 15(2):39-46. | NOT SELECTED. Only bottle feeding. |
| 1. Gaidhane AM, Patil M, Khatib N, Zodpey S, Zahiruddin QS. Prevalence and determinant of early childhood caries among the children attending the Anganwadis of Wardha district, India. Indian J Dent Res 2013; 24(2):199-205 | NOT SELECTED. Does not compare breastfeeding with bottle feeding. |
| 1. Tanaka K, Miyake Y, Sasaki S, Hirota Y. Infant feeding practices and risk of dental caries in Japan: the Osaka Maternal And Child Health Study. Pediatr Dent 2013; 35(3):267-271. | NOT SELECTED. Does not compare breastfeeding with bottle feeding. |
| 1. Bahuguna R, Younis KS, Jain A. Influence of feeding practices on dental caries. A case-control study. Eur J Paediatr Dent 2013; 14(1):55-58. | NOT SELECTED. Does not compare breastfeeding with bottle feeding. |
| 1. Folayan MO, Sowole CA, Kola-Jebutu A, Owotade FJ. Risk factors for rampant caries in children from southwestern Nigeria. Afr J Med Med Sci 2012; 41(3):249-255. | NOT SELECTED. Statistical data unavailable. |
| 1. Hong CH, Bagramian RA, Hashim Nainar SM, Straffon LH, Shen L, Hsu CY. High caries prevalence and risk factors among young preschool children in an urban community with water fluoridation. Int J Paediatr Dent 2014; 24(1):32-42. | NOT SELECTED. Only breastfeeding |
| 1. Masumo R, Bardsen A, Mashoto K, Astrom AN. Feeding practice among 6-36 months old in Tanzania and Uganda: reliability and relationship with early childhood caries, ECC. Acta Odontol Scand 2013; 71(5):1309-1318. | NOT SELECTED. Only breastfeeding. |
| 1. Sankeshwari RM, Ankola AV, Tangade PS, Hebbal MI. Feeding habits and oral hygiene practices as determinants of early childhood caries in 3- to 5-year-old children of Belgaum City, India. Oral Health Prev Dent 2012; 10(3):283-290. | NOT SELECTED. Does not compare breastfeeding with bottle feeding. |
| 1. Kramer MS, Kakuma R. Optimal duration of exclusive breastfeeding. Cochrane Database Syst Rev 2012; 8:CD003517. | NOT SELECTED. Only breastfeeding. |
| 1. Masumo R, Bardsen A, Mashoto K, Astrom AN. Prevalence and socio-behavioral influence of early childhood caries, ECC, and feeding habits among 6-36 months old children in Uganda and Tanzania. Bmc Oral Health 2012; 12:24. | NOT SELECTED. Only breastfeeding. |
| 1. Nunes AM, Alves CM, Borba de AF, Ortiz TM, Ribeiro MR, Silva AA et al. Association between prolonged breast-feeding and early childhood caries: a hierarchical approach. Community Dent Oral Epidemiol 2012; 40(6):542-549. | NOT SELECTED. Does not compare breastfeeding and bottle feeding. |
| 1. Retnakumari N, Cyriac G. Childhood caries as influenced by maternal and child characteristics in pre-school children of Kerala-an epidemiological study. Contemp Clin Dent 2012; 3(1):2-8. | NOT SELECTED. Does not compare breastfeeding and bottle feeding. |
| 1. Prakash P, Subramaniam P, Durgesh BH, Konde S. Prevalence of early childhood caries and associated risk factors in preschool children of urban Bangalore, India: A cross-sectional study. Eur J Dent 2012; 6(2):141-152. | NOT SELECTED. Does not compare breastfeeding and bottle feeding. |
| 1. Arora A, Bedros D, Bhole S, Do LG, Scott J, Blinkhorn A et al. Child and family health nurses' experiences of oral health of preschool children: a qualitative approach. J Public Health Dent 2012; 72(2):149-155. | NOT SELECTED. Does not compare breastfeeding and bottle feeding. |
| 1. Bankel M, Robertson A, Kohler B. Carious lesions and caries risk predictors in a group of Swedish children 2 to 3 years of age. One year observation. Eur J Paediatr Dent 2011; 12(4):215-219. | NOT SELECTED. Health promotion. |
| 1. Tanaka K, Miyake Y. Association between breastfeeding and dental caries in Japanese children. J Epidemiol 2012; 22(1):72-77. | NOT SELECTED. Only breastfeeding. |
| 1. Kumarihamy SL, Subasinghe LD, Jayasekara P, Kularatna SM, Palipana PD. The prevalence of Early Childhood Caries in 1-2 yrs olds in a semi-urban area of Sri Lanka. BMC Res Notes 2011; 4:336. | NOT SELECTED. Does not compare breastfeeding and bottle feeding. |
| 1. Ozer S, Sen TE, Bayrak S, Egilmez T. Evaluation of certain risk factors for early childhood caries in Samsun, Turkey. Eur J Paediatr Dent 2011; 12(2):103-106. | NOT SELECTED. Does not compare breastfeeding and bottle feeding |
| 1. Begzati A, Berisha M, Meqa K. Early childhood caries in preschool children of Kosovo - a serious public health problem. Bmc Public Health 2010; 10:788. | NOT SELECTED. Only bottle feeding. |
| 1. Feldens CA, Giugliani ER, Vigo A, Vitolo MR. Early feeding practices and severe early childhood caries in four-year-old children from southern Brazil: a birth cohort study. Caries Res 2010; 44(5):445-452. | NOT SELECTED. Does not compare breastfeeding and bottle feeding. |
| 1. Folayan MO, Sowole CA, Owotade FJ, Sote E. Impact of infant feeding practices on caries experience of preschool children. J Clin Pediatr Dent 2010; 34(4):297-301. | NOT SELECTED. Does not compare breastfeeding and bottle feeding. |
| 1. Johansson I, Holgerson PL, Kressin NR, Nunn ME, Tanner AC. Snacking habits and caries in young children. Caries Res 2010; 44(5):421-430. | NOT SELECTED. Feeding habits at night. |
| 1. Slabsinskiene E, Milciuviene S, Narbutaite J, Vasiliauskiene I, Andruskeviciene V, Bendoraitiene EA et al. Severe early childhood caries and behavioral risk factors among 3-year-old children in Lithuania. Medicina (Kaunas ) 2010; 46(2):135-141. | NOT SELECTED. Does not compare breastfeeding and bottle feeding. |
| 1. Feldens CA, Giugliani ER, Duncan BB, Drachler ML, Vitolo MR. Long-term effectiveness of a nutritional program in reducing early childhood caries: a randomized trial. Community Dent Oral Epidemiol 2010; 38(4):324-332. | NOT SELECTED. Health promotion – intervention programme. |
| 1. Jigjid B, Ueno M, Shinada K, Kawaguchi Y. Early childhood caries and related risk factors in Mongolian children. Community Dent Health 2009; 26(2):121-128. | NOT SELECTED. Does not compare breastfeeding and bottle feeding children. |
| 1. Aldy D, Siregar H, Liwijaya SG, Tanyati S. A comparative study of caries formation in breast-fed and bottle-fed children. Pediatr Indones 1979; 19(11-12): 308-312. | NOT SELECTED. Not found by COMUT. |
| 1. Mohebbi SZ, Virtanen JI, Vahid-Golpayegani M, Vehkalahti MM. Feeding habits as determinants of early childhood caries in a population where prolonged breastfeeding is the norm. Community Dent Oral Epidemiol 2008; 36(4):363-369. | NOT SELECTED. Does not compare breastfeeding and bottle feeding children. |
| 1. Tyagi R. The prevalence of nursing caries in Davangere preschool children and its relationship with feeding practices and socioeconomic status of the family. J Indian Soc Pedod Prev Dent 2008; 26(4):153-157. | NOT SELECTED. Night-time breastfeeding. |
| 1. Werneck RI, Lawrence HP, Kulkarni GV, Locker D. Early childhood caries and access to dental care among children of Portuguese-speaking immigrants in the city of Toronto. J Can Dent Assoc 2008; 74(9):805. | NOT SELECTED. Does not compare breastfeeding and bottle feeding children. |
| 1. White V. Breastfeeding and the risk of early childhood caries. Evid Based Dent 2008; 9(3):86-88. | NOT SELECTED. Systematic review. |
| 1. Mohamed N, Barnes J. Characteristics of children under 6 years of age treated for early chidhood caries in South Africa. J Clin Pediatr Dent 2008; 32(3):247-252. | NOT SELECTED. Statistical data unavailable. |
| 1. Qin M, Li J, Zhang S, Ma W. Risk factors for severe early childhood caries in children younger than 4 years old in Beijing, China. Pediatr Dent 2008; 30(2):122-128. | NOT SELECTED. Does not compare breastfeeding and bottle feeding children. |
| 1. Weber-Gasparoni K, Kanellis MJ, Levy SM, Stock J. Caries prior to age 3 and breastfeeding: a survey of La Leche League members. J Dent Child (Chic ) 2007; 74(1):52-61. | NOT SELECTED. Night-time feeding habits. |
| 1. Caplan LS, Erwin K, Lense E, Hicks J, Jr. The potential role of breast-feeding and other factors in helping to reduce early childhood caries. J Public Health Dent 2008; 68(4):238-241. | NOT SELECTED. Statistical data not available. |
| 1. Vazquez-Nava F, Vazquez RE, Saldivar GA, Beltran GF, Almeida AV, Vazquez RC. Allergic rhinitis, feeding and oral habits, toothbrushing and socioeconomic status. Effects on development of dental caries in primary dentition. Caries Res 2008; 42(2):141-147. | NOT SELECTED. Does not compare breastfeeding and bottle feeding children. |
| 1. Campus G, Solinas G, Sanna A, Maida C, Castiglia P. Determinants of ECC in Sardinian preschool children. Community Dent Health 2007; 24(4):253-256. | NOT SELECTED. Does not compare breastfeeding and bottle feeding children. |
| 1. Robke FJ. Effects of nursing bottle misuse on oral health. Prevalence of caries, tooth malalignments and malocclusions in North-German preschool children. J Orofac Orthop 2008; 69(1):5-19. | NOT SELECTED. Only bottle feeding. |
| 1. Barge K. Breast-feeding doesn't contribute to dental caries. J Dent Hyg 2007; 81(4):69. | NOT SELECTED. Review |
| 1. Nishimura M, Oda T, Kariya N, Matsumura S, Shimono T. Using a caries activity test to predict caries risk in early childhood. J Am Dent Assoc 2008; 139(1):63-71. | NOT SELECTED. Statistical data unavailable. |
| 1. Iida H, Auinger P, Billings RJ, Weitzman M. Association between infant breastfeeding and early childhood caries in the United States. Pediatrics 2007; 120(4):e944-e952. | NOT SELECTED. Does not compare breastfeeding and bottle feeding. |
| 1. Kramer MS, Vanilovich I, Matush L, Bogdanovich N, Zhang X, Shishko G et al. The effect of prolonged and exclusive breast-feeding on dental caries in early school-age children. New evidence from a large randomized trial. Caries Res 2007; 41(6):484-488. | NOT SELECTED. Children above 71 months. |
| 1. Tiberia MJ, Milnes AR, Feigal RJ, Morley KR, Richardson DS, Croft WG et al. Risk factors for early childhood caries in Canadian preschool children seeking care. Pediatr Dent 2007; 29(3):201-208. | NOT SELECTED. Different etiology: weaning. |
| 1. Livny A, Assali R, Sgan-Cohen HD. Early Childhood Caries among a Bedouin community residing in the eastern outskirts of Jerusalem. Bmc Public Health 2007; 7:167. | NOT SELECTED. Does not compare breastfeeding and bottle feeding. |
| 1. Yonezu T, Yotsuya K, Yakushiji M. Characteristics of breast-fed children with nursing caries. Bull Tokyo Dent Coll 2006; 47(4):161-165. | NOT SELECTED. Only breastfeeding. |
| 1. Yonezu T, Ushida N, Yakushiji M. Longitudinal study of prolonged breast- or bottle-feeding on dental caries in Japanese children. Bull Tokyo Dent Coll 2006; 47(4):157-160. | NOT SELECTED. Different etiology: weaning |
| 1. Ollila P, Larmas M. A seven-year survival analysis of caries onset in primary second molars and permanent first molars in different caries risk groups determined at age two years. Acta Odontol Scand 2007; 65(1):29-35. | NOT SELECTED. Does not compare breastfeeding children and bottle feeding children. |
| 1. Ersin NK, Eronat N, Cogulu D, Uzel A, Aksit S. Association of maternal-child characteristics as a factor in early childhood caries and salivary bacterial counts. J Dent Child (Chic ) 2006; 73(2):105-111. | NOT SELECTED. In vitro study |
| 1. Spitz AS, Weber-Gasparoni K, Kanellis MJ, Qian F. Child temperament and risk factors for early childhood caries. J Dent Child (Chic ) 2006; 73(2):98-104. | NOT SELECTED. Different etiology: children temperament |
| 1. Martens L, Vanobbergen J, Willems S, Aps J, De MJ. Determinants of early childhood caries in a group of inner-city children. Quintessence Int 2006; 37(7):527-536. | NOT SELECTED. Does not compare breastfeeding and bottle feeding children. |
| 1. Hallett KB, O'Rourke PK. Pattern and severity of early childhood caries. Community Dent Oral Epidemiol 2006; 34(1):25-35. | NOT SELECTED. Does not compare breastfeeding and bottle feeding children. |
| 1. Moura LF, de Moura MS, de Toledo OA. Dental caries in children that participated in a dental program providing mother and child care. J Appl Oral Sci 2006; 14(1):53-60. | NOT SELECTED. Children above 71 months old. |
| 1. van Palenstein Helderman WH, Soe W, van 't Hof MA. Risk factors of early childhood caries in a Southeast Asian population. J Dent Res 2006; 85(1):85-88. | NOT SELECTED. Only breastfeeding. |
| 1. Singh P, King T. Infant and child feeding practices and dental caries in 6 to 36 months old children in Fiji. Pac Health Dialog 2003; 10(1):12-16. | NOT SELECTED. Does not compare breastfeeding and bottle feeding children. |
| 1. Schroth RJ, Moffatt ME. Determinants of early childhood caries (ECC) in a rural Manitoba community: a pilot study. Pediatr Dent 2005; 27(2):114-120. | NOT SELECTED. Does not compare breastfeeding and bottle feeding children. |
| 1. Zeng X, Luo Y, Du M, Bedi R. Dental caries experience of preschool children from different ethnic groups in Guangxi Province in China. Oral Health Prev Dent 2005; 3(1):25-31. | NOT SELECTED. Does not compare breastfeeding and bottle feeding children. |
| 1. Azevedo TD, Bezerra AC, de Toledo OA. Feeding habits and severe early childhood caries in Brazilian preschool children. Pediatr Dent 2005; 27(1):28-33. | NOT SELECTED. Different etiology: nocturnal breastfeeding and bottle during the day. |
| 1. Sayegh A, Dini EL, Holt RD, Bedi R. Oral health, sociodemographic factors, dietary and oral hygiene practices in Jordanian children. J Dent 2005; 33(5):379-388. | NOT SELECTED. Does not compare breastfeeding and bottle feeding children. |
| 1. Schroth RJ, Smith PJ, Whalen JC, Lekic C, Moffatt ME. Prevalence of caries among preschool-aged children in a northern Manitoba community. J Can Dent Assoc 2005; 71(1):27. | NOT SELECTED. Statistical data unavailable. |
| 1. Rosenblatt A, Zarzar P. Breast-feeding and early childhood caries: an assessment among Brazilian infants. Int J Paediatr Dent 2004; 14(6):439-445. | NOT SELECTED. Different etiology: bottle content. |
| 1. Gaffney KE, Farrar-Simpson MA, Claure D, Davilla G. Prolonged baby bottle feeding: a health risk factor. Pediatr Nurs 2004; 30(3):242-245. | NOT SELECTED. Review/Case report. |
| 1. Vachirarojpisan T, Shinada K, Kawaguchi Y, Laungwechakan P, Somkote T, Detsomboonrat P. Early childhood caries in children aged 6-19 months. Community Dent Oral Epidemiol 2004; 32(2):133-142. | NOT SELECTED. Statistical data unavailable. |
| 1. Bray KK, Branson BG, Williams K. Early childhood caries in an urban health department: an exploratory study. J Dent Hyg 2003; 77(4):225-232. | NOT SELECTED. Statistical data unavailable. |
| 1. King NM, Wu II, Tsai JS. Caries prevalence and distribution, and oral health habits of zero- to four-year-old children in Macau, China. J Dent Child (Chic ) 2003; 70(3):243-249. | NOT SELECTED. Does not compare breastfeeding and bottle feeding. |
| 1. Dye BA, Shenkin JD, Ogden CL, Marshall TA, Levy SM, Kanellis MJ. The relationship between healthful eating practices and dental caries in children aged 2-5 years in the United States, 1988-1994. J Am Dent Assoc 2004; 135(1):55-66. | NOT SELECTED. Only bottle feeding. |
| 1. Jose B, King NM. Early childhood caries lesions in preschool children in Kerala, India. Pediatr Dent 2003; 25(6):594-600. | NOT SELECTED. Does not compare breastfeeding and bottle feeding. |
| 1. Olmez S, Uzamis M, Erdem G. Association between early childhood caries and clinical, microbiological, oral hygiene and dietary variables in rural Turkish children. Turk J Pediatr 2003; 45(3):231-236. | NOT SELECTED. In vitro study. |
| 1. Jin BH, Ma DS, Moon HS, Paik DI, Hahn SH, Horowitz AM. Early childhood caries: prevalence and risk factors in Seoul, Korea. J Public Health Dent 2003; 63(3):183-188. | NOT SELECTED. Does not compare breastfeeding and bottle feeding. |
| 1. Carino KM, Shinada K, Kawaguchi Y. Early childhood caries in northern Philippines. Community Dent Oral Epidemiol 2003; 31(2):81-89. | NOT SELECTED. Children above 71 months old. |
| 1. Rosenblatt A, Zarzar P. The prevalence of early childhood caries in 12- to 36-month-old children in Recife, Brazil. ASDC J Dent Child 2002; 69(3):319-24, 236. | NOT SELECTED. Statistical data unavailable. |
| 1. Huntington NL, Kim IJ, Hughes CV. Caries-risk factors for Hispanic children affected by early childhood caries. Pediatr Dent 2002; 24(6):536-542. | NOT SELECTED. Does not compare breastfeeding and bottle feeding. |
| 1. Hallett KB, O'Rourke PK. Early childhood caries and infant feeding practice. Community Dent Health 2002; 19(4):237-242. | NOT SELECTED. Does not compare breastfeeding and bottle feeding. |
| 1. Olmez S, Uzamris M. Risk factors of early childhood caries in Turkish children. Turk J Pediatr 2002; 44(3):230-236. | NOT SELECTED. Does not compare breastfeeding and bottle feeding. |
| 1. Santos AP, Soviero VM. Caries prevalence and risk factors among children aged 0 to 36 months. Pesqui Odontol Bras 2002; 16(3):203-208. | NOT SELECTED. Night-time breastfeeding. |
| 1. Chan SC, Tsai JS, King NM. Feeding and oral hygiene habits of preschool children in Hong Kong and their caregivers' dental knowledge and attitudes. Int J Paediatr Dent 2002; 12(5):322-331. | NOT SELECTED. Does not compare breastfeeding and bottle feeding. |
| 1. Ngatia EM, Imungi JK, Muita JW, Nganga PM. Dietary patterns and dental caries in nursery school children in Nairobi, Kenya. East Afr Med J 2001; 78(12):673-677 | NOT SELECTED. Only bottle feeding. |
| 1. Lulic-Dukic O, Juric H, Dukic W, Glavina D. Factors predisposing to early childhood caries (ECC) in children of pre-school age in the city of Zagreb, Croatia. Coll Antropol 2001; 25(1):297-302. | NOT SELECTED. Only bottle feeding. |
| 1. Fraiz FC, Walter LR. Study of the factors associated with dental caries in children who receive early dental care. Pesqui Odontol Bras 2001; 15(3):201-207. | NOT SELECTED. Only bottle feeding. |
| 1. Wyne A, Darwish S, Adenubi J, Battata S, Khan N. The prevalence and pattern of nursing caries in Saudi preschool children. Int J Paediatr Dent 2001; 11(5):361-364. | NOT SELECTED. Only bottle feeding. |
| 1. Tsai AI, Johnsen DC, Lin YH, Hsu KH. A study of risk factors associated with nursing caries in Taiwanese children aged 24-48 months. Int J Paediatr Dent 2001; 11(2):147-149. | NOT SELECTED. Does not study breastfeeding and bottle feeding. |
| 1. Tada A, Ando Y, Hanada N. Caries risk factors among three-year old children in Chiba, Japan. Asia Pac J Public Health 1999; 11(2):109-112. | NOT SELECTED. Does not compare breastfeeding and bottle feeding. |
| 1. Douglass JM, Tinanoff N, Tang JM, Altman DS. Dental caries patterns and oral health behaviors in Arizona infants and toddlers. Community Dent Oral Epidemiol 2001; 29(1):14-22. | NOT SELECTED. Night-time habits. |
| 1. Petti S, Cairella G, Tarsitani G. Rampant early childhood dental decay: an example from Italy. J Public Health Dent 2000; 60(3):159-166. | NOT SELECTED. Does not compare breastfeeding and bottle feeding. |
| 1. Milgrom P, Riedy CA, Weinstein P, Tanner AC, Manibusan L, Bruss J. Dental caries and its relationship to bacterial infection, hypoplasia, diet, and oral hygiene in 6- to 36-month-old children. Community Dent Oral Epidemiol 2000; 28(4):295-306. | NOT SELECTED. Does not compare breastfeeding and bottle feeding. |
| 1. Dini EL, Holt RD, Bedi R. Caries and its association with infant feeding and oral health-related behaviours in 3-4-year-old Brazilian children. Community Dent Oral Epidemiol 2000; 28(4):241-248. | NOT SELECTED. Does not compare breastfeeding and bottle feeding. |
| 1. Ismail AI, Sohn W. A systematic review of clinical diagnostic criteria of early childhood caries. J Public Health Dent 1999; 59(3):171-191. | NOT SELECTED. Review |
| 1. Oulis CJ, Berdouses ED, Vadiakas G, Lygidakis NA. Feeding practices of Greek children with and without nursing caries. Pediatr Dent 1999; 21(7):409-416. | NOT SELECTED. Does not compare breastfeeding and bottle feeding. |
| 1. al Ghanim NA, Adenubi JO, Wyne AA, Khan NB. Caries prediction model in pre-school children in Riyadh, Saudi Arabia. Int J Paediatr Dent 1998; 8(2):115-122. | NOT SELECTED. Does not compare breastfeeding and bottle feeding. |
| 1. Mattos-Graner RO, Zelante F, Line RC, Mayer MP. Association between caries prevalence and clinical, microbiological and dietary variables in 1.0 to 2.5-year-old Brazilian children. Caries Res 1998; 32(5):319-323. | NOT SELECTED. Does not compare breastfeeding and bottle feeding. |
| 1. Wyne AH, Adenubi JO, Shalan T, Khan N. Feeding and socioeconomic characteristics of nursing caries children in a Saudi population. Pediatr Dent 1995; 17(7):451-454. | NOT SELECTED. Statistical data unavailable. |
| 1. Hallonsten AL, Wendt LK, Mejare I, Birkhed D, Hakansson C, Lindvall AM et al. Dental caries and prolonged breast-feeding in 18-month-old Swedish children. Int J Paediatr Dent 1995; 5(3):149-155. | NOT SELECTED. Only breastfeeding. |
| 1. Roberts GJ, Cleaton-Jones PE, Fatti LP, Richardson BD, Sinwel RE, Hargreaves JA et al. Patterns of breast and bottle feeding and their association with dental caries in 1- to 4-year-old South African children. 1. Dental caries prevalence and experience. Community Dent Health 1993; 10(4):405-413. | NOT SELECTED. Statistical data unavailable. |
| 1. Serwint JR, Mungo R, Negrete VF, Duggan AK, Korsch BM. Child-rearing practices and nursing caries. Pediatrics 1993; 92(2):233-237. | NOT SELECTED. Does not compare breastfeeding and bottle feeding. |
| 1. Babeely K, Kaste LM, Husain J, Behbehani J, al-Za'abi F, Maher TC et al. Severity of nursing-bottle syndrome and feeding patterns in Kuwait. Community Dent Oral Epidemiol 1989; 17(5):237-239. | NOT SELECTED. Does not compare breastfeeding and bottle feeding. |
| 1. Broderick E, Mabry J, Robertson D, Thompson J. Baby bottle tooth decay in Native American children in Head Start centers. Public Health Rep 1989; 104(1):50-54. | NOT SELECTED. Does not compare breastfeeding and bottle feeding. |
| 1. Schroth RJ, Halchuk S, Star L. Prevalence and risk factors of caregiver reported Severe Early Childhood Caries in Manitoba First Nations children: results from the RHS Phase 2 (2008-2010). Int J Circumpolar Health. 2013 Aug 5;72. | NOT SELECTED. Does not compare breastfeeding and bottle feeding. |
